# A 50-year record of NO_x _and SO_2 _sources in precipitation in the Northern Rocky Mountains, USA

**DOI:** 10.1186/1467-4866-12-4

**Published:** 2011-03-07

**Authors:** David L Naftz, Paul F Schuster, Craig A Johnson

**Affiliations:** 1 U.S. Geological Survey, 2329 W. Orton Circle, Salt Lake City, UT 84119, USA; 2 U.S. Geological Survey, 3215 Marine Street, Suite E-127, Boulder, CO 80303, USA; 3 U.S. Geological Survey, Denver Federal Center, MS 963, Denver, CO 80225, USA

## Abstract

Ice-core samples from Upper Fremont Glacier (UFG), Wyoming, were used as proxy records for the chemical composition of atmospheric deposition. Results of analysis of the ice-core samples for stable isotopes of nitrogen (δ^15^N, ) and sulfur (δ^34^S, ), as well as  and  deposition rates from the late-1940s thru the early-1990s, were used to enhance and extend existing National Atmospheric Deposition Program/National Trends Network (NADP/NTN) data in western Wyoming. The most enriched δ^34^S value in the UFG ice-core samples coincided with snow deposited during the 1980 eruption of Mt. St. Helens, Washington. The remaining δ^34^S values were similar to the isotopic composition of coal from southern Wyoming. The δ^15^N values in ice-core samples representing a similar period of snow deposition were negative, ranging from -5.9 to -3.2 ‰ and all fall within the δ^15^N values expected from vehicle emissions. Ice-core nitrate and sulfate deposition data reflect the sharply increasing U.S. emissions data from 1950 to the mid-1970s.

## Introduction

**Figure 1:**
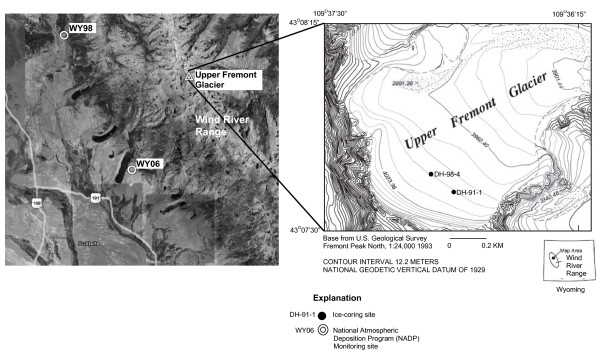
**Location of NADP/NTN and ice-coring sites in Wind River Range, Wyoming**.

The chemical quality of snowfall deposited in high-elevation areas in the Rocky Mountain region can be affected by energy generation and associated population growth [1,2]. High elevation areas in the Wind River Range (WRR) of Wyoming (figure 1) exceed 4 km above sea level and are adjacent to areas of accelerating energy development [3]. For example, over 3,000 natural gas wells are being installed in the Green River Basin, directly west of the WRR. Full development of the Jonah gas field could result in the production of 1,480 metric tons/yr of NO_x _and 25.7 metric tons/yr of SO_2 _[3].

Thin soils and dilute surface-water systems in high-elevation areas have limited capacities to buffer increased acidity associated with the airborne contaminants of NO_x _and SO_2_. Trends in precipitation chemistry at NADP/NTN sites in the western United States have indicated an increase in total N deposition and a decrease in  deposition from 1981-1998 [4]. In addition to monitoring trends in N and S deposition, the isotopic composition of snow, firn, and ice has been used to differentiate natural and anthropogenic solute sources. Stable isotope ratios of sulfur in , expressed as δ^34^S, were monitored in bulk snowpack samples collected from a network of 52 high-elevation sites in the Rocky Mountains from 1993 to 1999 [5]. The δ^34^S values indicated that snowpack S in high-elevation areas is primarily derived from anthropogenic sources [5].

Changes in δ^34^S values in firn and ice-core samples have been used to reconstruct changes in sulfate sources to central Asia, Greenland, and Antarctica. The variation in δ^34^S values in a firn core from central Asia allowed for the identification of S derived from marine evaporites (+15 ‰) during high dust deposition events and anthropogenic emissions (+5.4 ‰) [6]. Preindustrial δ^34^S signatures in Greenland ice cores were comprised of marine biogenic emissions, continental dust sources, background volcanism, and continental biota [7]. Beginning in 1870 A.D., δ^34^S signatures in the ice-core samples indicated anthropogenic S sources.

In Antarctica, shallow firn cores collected from the South Pole contained δ^34^S values that were used to confirm and contrast the different S isotopic signals between the low-latitude Agunge volcanic eruption in 1963 and background marine biogenic sulfate [8]. Composited ice-core samples representing coastal and plateau regions on the Antarctic ice sheet were found to have similar δ^34^S values over the past 1,100 years, indicative of no temporal change in influencing sources of S [9]. Values of δ^34^S in two ice cores from east Antarctica over the complete glacial/interglacial cycle were significantly lighter than previous measurements of δ^34^S from the South Pole [10]. A likely mechanism for the observed isotopic difference was Rayleigh-type fractionation as S species are oxidized and transported toward the East Antarctic Plateau [10]. Measurements of δ^34^S values in an ice core collected from west Antarctica were found to be a mixture of marine and volcanic S sources during the time period from 1935-1976. [11].

Stable isotopes have also been used to gain a better understanding of N sources in atmospheric deposition. Historical records of the isotopic composition of N_2_O in trapped gases (ice cores) from Greenland and interstitial air (snowpack) from the South Pole have been used to differentiate between natural and anthropogenic sources [12]. The δ^15^N value of atmospheric N_2_O has dropped by 1.7 ‰ during the 20^th ^century, likely due to increasing agricultural activities [12]. In support of these measurements, simulations of N_2_O_(g) _have indicated a -1.8 ‰ shift in δ^15^N over the last two centuries, primarily due to anthropogenic influences [13]. The δ^15^N of  in wet deposition from 33 sites in the northeastern United States was strongly correlated with the location of coal-fired powerplants [14]. Ice-core samples from Greenland indicated decreasing δ^15^N () values with increasing  concentrations; however, more information is needed to confirm this trend and differentiate source area vs. post-depositional processes during the firn-to-ice transition [15]. A more recent ice-core record from Greenland, spanning deposition from 1718 to 2006, revealed a clear trend of decreasing δ^15^N () values from 11 ‰ (pre-industrial) to -1 ‰ (~1996-2006) [16].

Although previous studies have shown that δ^15^N () values of snow and ice samples have excellent potential for providing information on  sources, post-depositional changes in the concentration and isotopic composition of  needs to be considered [17,18]. Year-round measurement of δ^15^N () values in snow pits from Dome C, Antarctica, indicated strong enrichment relative to atmospheric  and loss of  mass from the snow surface due to UV-photolysis [17]. In contrast to the Antarctica results, a similar study in Greenland [18] indicated minimal influence of photolysis on the isotopic composition of δ^15^N () in firn and ice. One possible reason for the different research results between the two studies may be the higher snow accumulation rates at the Greenland study site [18]. Higher rates of snow accumulation were found to mitigate the magnitude of post-depositional processing and loss of  in the snowpack [19].

Glaciers present in the high elevation regions of the WRR, Wyoming, present a unique opportunity to couple short-term (1980 to present) NADP/NTN data (sites WY06 and WY98) trends in the chemistry of atmospheric deposition with similar and longer term data preserved in glacial ice from the nearby UFG (figure 1). The UFG is the only glacier within the continental U.S. where ice cores have been documented to contain paleoenvironmental and paleoclimatological records [20-27]. Characteristics present at UFG conducive to preserving paleonvironmental signals include: (1) ice-coring site altitudes that exceed 4 km above sea level (ASL) to minimize meltwater modification of the snow and ice chemistry and (2) large ice thicknesses (ranging from 60 to 172 m in the upper half of the glacier) to provide long-term paleoenvironmental records.

Ice cores exceeding 160 m in length were recovered from UFG in 1991 and 1998 [23,24]. The 1991 ice core was estimated to contain 250 years of record as determined from carbon-14 dating of an insect leg recovered from near the bottom of the core [23]. This length of record was subsequently confirmed by a higher-resolution chronology established with continuous electrical conductivity measurements [26]. On the basis of these data, ice at the bottom of UFG in proximity to the 1991 and 1998 drilling sites was likely deposited as snow prior to 1710 AD.

The overall objective of this paper is to demonstrate the effectiveness of using the isotopic composition and concentration of  and  in ice-core samples collected from the UFG to supplement and extend existing NADP/NTN records. Specific objectives are to: (1) determine atmospheric deposition sources since the late 1940s by determining the isotopic values of δ^34^S () and δ^15^N () in ice-core samples; (2) reconstruct changes in  and  wet deposition rates from ice-core samples representative of snow deposited from the early-1950s to early-1990s; and (3) couple the results from objectives 1 and 2 with existing NADP/NTN deposition records and NO_X_/SO_2 _emission records to identify processes controlling long-term trends in the loading of S and N at high-elevation sites.

## Methods

### Field

Ice cores were collected from UFG in 1991 and 1998 using a thermal drill as described in [23]. The ice cores were collected in 1- and 2-m segments. On-site processing of the cores included visual inspection, logging, and density determinations by personnel wearing Tyvek suits and powder-free Latex gloves. The cores were sealed in polyethylene bags, placed in plastic core tubes, and stored in snow vaults until removal from the site to a freezer truck via a 10-minute helicopter flight. The UFG ice cores are currently archived at the National Ice Core Laboratory (NICL) in Lakewood, Colorado.

### Laboratory

Ice-core samples were melted according to strict protocols [21] to minimize sample contamination. Ice cores were subsampled using a bandsaw frequently cleaned with ethanol in cold room laboratories at NICL. Multiple core sections from each interval were composited in order to obtain sufficient S and N mass for isotopic analyses. The surface ice from each subsample was scraped away with a stainless steel microtome. Each ice sample was thoroughly rinsed with ultrapure (18.0 megaohm) deionized water and placed in a prerinsed and covered plastic container. Each sample was allowed to melt at room temperature for one hour (or until approximately 15 mL of meltwater had accumulated). After this initial melt period the sample was rinsed in the accumulated meltwater, and the melt was discarded. The remaining sample was allowed to melt in the covered plastic container at room temperature.

For S isotopic analysis, meltwater volumes corresponding to 90-100 micrograms of sulfate (0.5-6 L) were evaporated to dryness within covered canisters flushed by dry nitrogen. The samples were transferred from 4-L Pyrex beakers to smaller Savillex beakers when they had been reduced to about 100 mL. Drydown salts were redissolved in a few mL of deionized water which was pipetted into tin capsules and evaporated a few hundred microliters at a time. One to 2 mg of powdered V_2_O_5 _was added and the capsules were pinched closed.

Filled capsules were combusted in an elemental analyzer to produce SO_2 _for analysis by isotope ratio monitoring using a Micromass Optima [28]. The elemental analyzer-mass spectrometer system was standardized using reference materials previously calibrated against IAEA-SO-5 and NBS 127 assuming δ^34^S values for them of -34.05 and 21.1 ‰, respectively [29]. To evaluate accuracy, synthetic Ca^2+^-Na^+^-K^+ ^solutions were prepared with reagents of known isotopic composition and then dried and analyzed. Sodium-dominated solutions gave δ^34^S values indistinguishable from the reagents used to prepare them; however, solutions with Na^+^:Ca^2+ ^ratios equivalent to the UFG samples were about 1 ‰ low. The cause of this inaccuracy is uncertain. No attempt was made to correct the UFG measurements for a possible similar inaccuracy. The S isotopic compositions are reported in δ-notation relative to Vienna Canon Diablo troilite. Reproducibility for triplicate analysis of sample ISO-1 was ±0.4 ‰ (one standard deviation).

For N isotopic analysis, meltwater volumes corresponding to 1.2 micrograms of nitrate (6-80 mL) were evaporated to <5 mL by the same method used for sulfur. Nitrous oxide (N_2_O) was produced by the denitrifier method using the *Pseudomonas chlororaphis *bacterium (ATCC 43928) [30,31], and the isotope ratios were measured using a ThermoFinnigan Delta Plus XL equipped with an autosampler and gas bench device. To calibrate the measurements and correct for media blanks, aliquots of the reference materials USGS32 KNO_3 _(δ^15^N = 180 ‰), USGS34 KNO_3 _(δ^15^N = -1.8 ‰), and USGS35 NaNO_3 _(δ^15^N = 2.7 ‰) were analyzed along with the unknowns.

Because the  in UFG samples is atmospheric in origin, it is likely to contain excess ^17^O (commonly expressed as values of Δ^17^O greater than zero) [32]. For single bacterium experiments like those carried out in this study, excess ^17^O leads to δ^15^N results that are erroneously high by 1 ‰ per 18.8 ‰ increase in Δ^17^O [33]. Our δ^15^N determinations for USGS35, which has a Δ^17^O value of 21.1 ‰, showed an error corresponding to 1 ‰ per 18.3 ‰ increase in Δ^17^O. The highest Δ^17^O values that have been observed to date in modern and ancient atmospheric nitrate are 39 and 20 ‰, respectively [34-36], and the results of a global model for the modern atmosphere suggest that Δ^17^O of atmospheric nitrate is unlikely to exceed about 30 ‰ in the western United States [37]. If the  in UFG samples has Δ^17^O values of 30 ‰ or lower, the error in our δ^15^N results is no more than 1.6 ‰. The N isotopic compositions are reported in δ-notation relative to atmospheric N_2(g)_. Reproducibility for triplicate analysis of USGS34 was ±0.27‰ (one standard deviation).

After filtration with 0.45 μm disposable polysulfone filters, a modified ion chromatography procedure for low ionic strength samples was used for the analysis of  and [38]. A Dionex DX-120 was configured with a 250 μL loop to increase instrument sensitivity. Ten percent of each sample batch consisted of quality control standards. Seven-point calibration curves covering the range of solutes being measured were run daily. Each batch contained sets of blanks (18.2 megaohm deionized water) and a series of USGS standard water reference samples (SRWS) at intervals of approximately every 10 ice-core samples. SRWS and blanks were monitored during sample runs. If the SRWS were not within 10% of expected values or the blanks were above detection limits, the sample run results were rejected and repeated.

All chemical data from the ice-core analyses can be found in additional file 1.

## Results and Discussion

### Ice-core chronology

**Figure 2:**
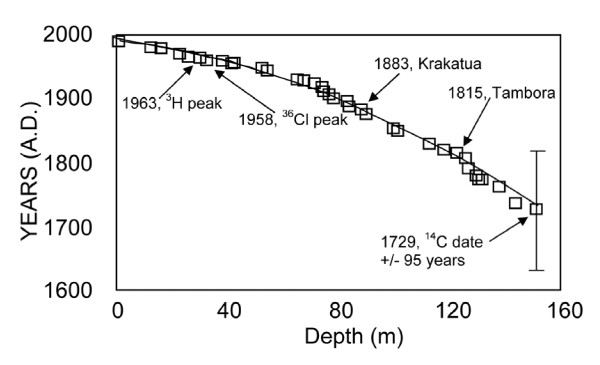
**Plot of reported volcanic events and isotopic age dates used to generate a polynomial fit for an age-depth profile of the Upper Fremont Glacier ice core collected in 1991 **[26]**, Wind River Range, Wyoming**. A slightly modified age-depth profile was developed for the 1998 ice core (DH-98-4) to account for the additional snow deposited between 1991 and 1998.

Tritium, chloride-36, and carbon-14 age-dating methods [23,39] were combined with the timing of major volcanic eruption signals that were evident in the core [26] to establish an ice-core chronology. The polynomial fit for the age-depth profile in the ice core [26] was determined to be(1)

where D is depth below glacier surface, in m. This age-depth profile is in excellent agreement with known time horizons in the ice core that include (1) 1963 tritium and 1958 chloride-36 peaks from above-ground nuclear testing; (2) 1883 Krakatau volcanic eruption; (3) 1815 Tambora volcanic eruption; and (4) 1729 (± 95 years) carbon-14 age date of an insect leg entrapped in the ice (figure 2).

### Variation of δ^34^S in ice

**Figure 3:**
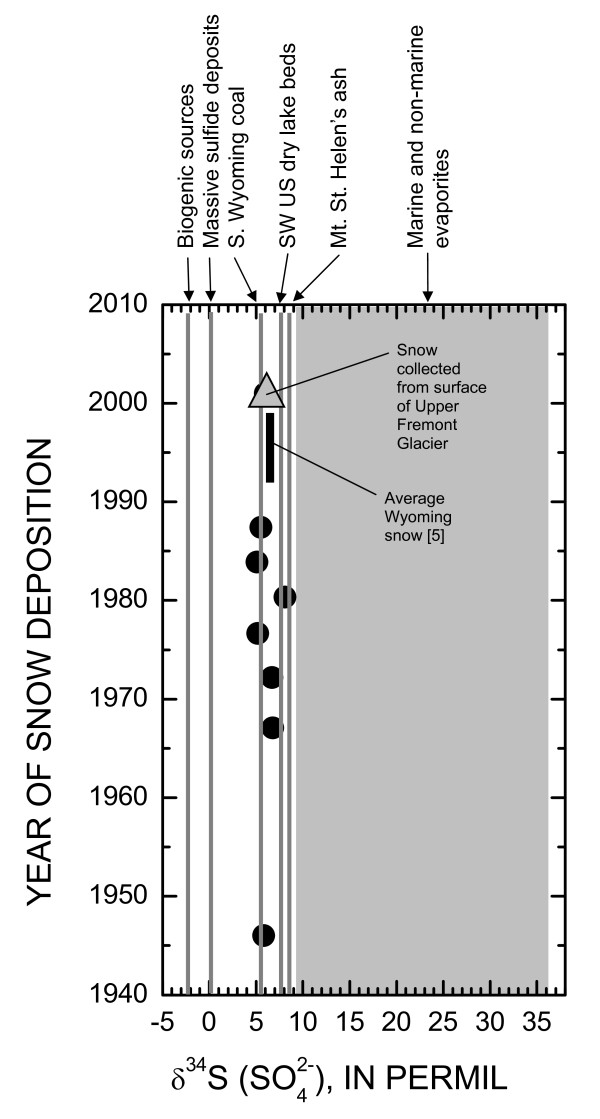
**Comparison of δ^34^S in ice-core and snow samples collected from Upper Fremont Glacier with δ^34^S values of potential sulfate sources to the Wind River Range, Wyoming**. Data for comparisons compiled from [5,40-45].

Seven composite ice-core samples and one snow sample collected from UFG were analyzed for δ^34^S (figure 3). The ice-core samples represent a time period from approximately 1946 to 1988 and δ^34^S values in the ice ranged from 5.1 to 8.1 ‰. Sulfate in snow and corresponding glacier ice can be derived from a variety of natural and anthropogenic sources such as sea-salt aerosols, biogenic emissions, entrained dust particles, fossil fuel combustion, and metal smelting. Volcanic eruptions can also provide an intermittent short-term source of atmospheric . The highest δ^34^S value in the UFG ice-core samples was associated with snow deposited primarily during 1980, coinciding with the eruption of Mt. St. Helens, Washington, during May 1980. Comparison of the δ^34^S value in the ice core (8.1 ‰) with a mean δ^34^S value of volcanic ash from the 1980 eruption of Mt. St. Helens (8.3 ‰) [40] indicates a similar isotopic composition. Corresponding geochemical signatures of the 1980 Mt. St. Helens volcanic eruption in the UFG ice-core samples include elevated Hg concentrations [27] and elevated electrical conductivity, , and Cl^- ^[26]. Ice samples collected from a 1980 deposition horizon on Knifepoint Glacier, approximately 4 km southwest of UFG, contained ash material that had morphology and chemical composition similar to Mt. St. Helens ash [20].

Except for the elevated δ^34^S value in the layer corresponding to atmospheric deposition from Mt. St. Helens, the remaining δ^34^S values in the ice-core samples ranged from 5.1 to 6.8 ‰ (figure 3). The δ^34^S values in the UFG ice core were substantially enriched relative to biogenic  sources (-2.4 ‰) [41] and massive sulfide deposits in Arizona (0 ‰) [42]. The δ^34^S values of massive sulfide deposits were assumed to be representative of smelter emissions associated with ore processing in Arizona and New Mexico [5]. Marine aerosols, which have a δ^34^S value of +20 ‰ [5], could not be a large source of  to UFG due to the much lighter δ^34^S isotopic values found in the ice.

The WRR and UFG are in close proximity to large expanses of arid and semi-arid lands composed of soils, sedimentary rocks, and dry lakebeds containing gypsum and anhydrite [5]. Therefore, dry deposition of dust on the surface of UFG during the summer and dust solute sources associated with snow deposition could comprise a large natural  source and corresponding isotopic signal. Marine and non-marine evaporites have δ^34^S values that range from +9 to +33 ‰ [43] and are much heavier relative to δ^34^S values found in UFG ice-core samples (figure 3). Median δ^34^S values of water soluble  from dry lake beds in arid regions of the southwestern US are +7.5 ‰ [44], slightly enriched relative to non-volcanic δ^34^S values in the UFG ice core. Based on the isotopic composition of snowpack  samples collected from high-elevation sites in northern New Mexico and southern Colorado from 1993 to 1999, it was concluded that soils and lakebeds were not dominant  sources [5]. The mean δ^34^S of snow collected from Wyoming during 1993-1999 had the same δ^34^S as a snow sample collected from the surface of UFG in 2001 (figure 3). This suggests that soils and lakebeds are not dominant  sources; however, two of the δ^34^S samples (mid-1960s and mid-1970s) may reflect contributions from lake-bed sulfate.

Coal from southern Wyoming, commonly used in electricity-producing power plants in close proximity to UFG, has a mean δ^34^S composition of 5.1 ‰ for organic S [45], similar in isotopic composition to the δ^34^S values in ice-core samples from UFG (figure 3). Assuming limited isotopic fractionation during coal combustion, the δ^34^S isotopic composition in the ice-core samples indicates that coal-fired powerplant emissions could be a dominant source of S deposition to high-elevation areas of the WRR. Based on U.S. emissions data compiled for 1998, electric utilities contributed the majority of SO_2 _emissions, representing 68% of total national SO_2 _emissions [46]. Coal combustion comprised over 90% of electric utility emissions on a national scale.

### Variation of δ^15^N in ice

**Figure 4:**
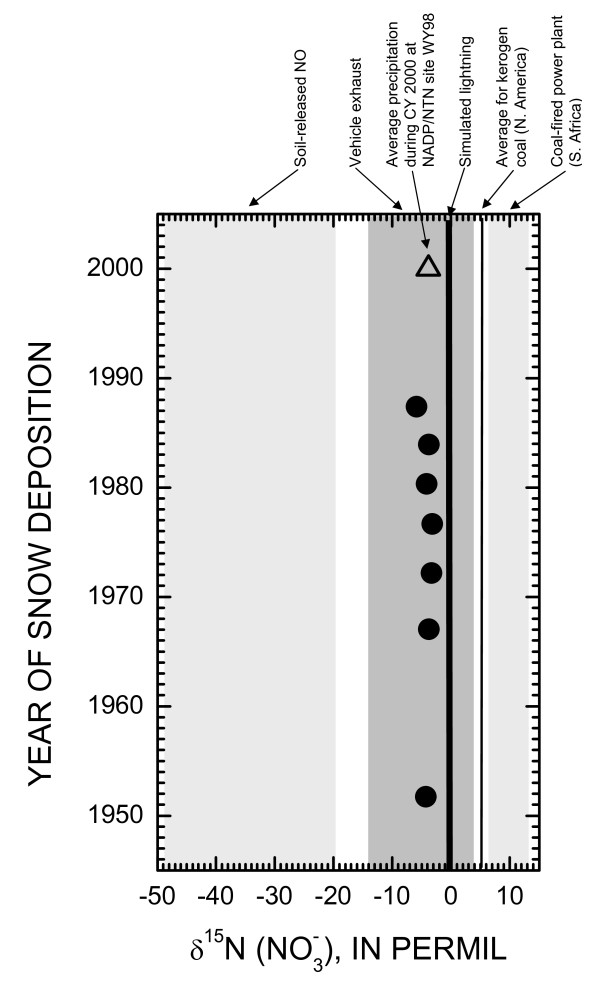
**Comparison of δ^15^N in ice-core samples collected from Upper Fremont Glacier with δ^15^N values of potential nitrate sources and site-specific data collected from Wyoming NADP/NTN site WY98**. Data for comparisons compiled from [47-50], and C. Kendall, USGS, written commun., 2008.

Seven composite ice-core samples representing the time period of deposition from 1951 to 1988 were collected from UFG and analyzed for δ^15^N in  (figure 4). The δ^15^N values in the ice were all negative and had similar isotopic composition, ranging from -5.9 to -3.2 ‰. Nitrate in snow and corresponding glacier ice can be derived from a variety of natural (lightning, biogenic soil processes, and wildfires) and anthropogenic sources (generation of electricity, industrial processes, and vehicle exhaust) [14]. The mean δ^15^N value in the UFG ice-core samples (-4.0 ‰, n = 7) was equal to the mean δ^15^N value of six archived precipitation samples collected during 2000 at NADP/NTN site WY98 (C. Kendall, U.S. Geological Survey, written commun., 2008). The similarity in mean δ^15^N values between the ice-core and NADP samples suggests similar  source(s).

Ice-core δ^15^N values were compared with δ^15^N values from potential NO_X _sources from previously published data (figure 4). Although δ^15^N values from coal deposits in the western U.S. were not available, ice-core δ^15^N values were substantially lighter than δ^15^N values of NO_X _from coal-fired powerplants in South Africa [47], simulated lightning [48], or kerogen in North American coal deposits [49]. Ice-core δ^15^N values were significantly enriched relative to soil-released biogenic NO values [50]. The δ^15^N of the ice-core samples all fall within the δ^15^N values expected from vehicle emissions [47,50]. Based on national NO_X _emission data compiled by the U.S. Environmental Protection Agency [46], on- and off-road vehicle emissions comprised 53% of NO_X _emissions during 1998 as opposed to electrical generation (25%) and industrial combustion (12%). Data compiled for the western United States indicate that on- and off-road mobile sources comprise 67% of the anthropogenic NO_x _emissions, with stationary sources comprising only 22% of the anthropogenic NO_x _emissions [51]. Based on the comparisons of the tightly constrained UFG ice-core δ^15^N values with existing end-member data, it appears likely that vehicular NO_X _emissions may have a large impact on  sources in snowfall deposited in the WRR of Wyoming.

The ice-core results conflict with a recent study in the more densely populated eastern United States where it was found that NO_x _emissions from stationary sources were strongly correlated with spatial variation of δ^15^N () in wet deposition [14]. One possible reason for the observed discrepancy with the ice-core results could be the lower number of stationary NO_x _sources in the western United States to support the lower population density. Additional δ^15^N values of NO_X _emissions from regional biogenic sources, as well as natural gas production facilities and coal-fired power plants in the western U.S. are needed to better assess  sources.

### Reconstruction of  and  deposition

**Figure 5:**
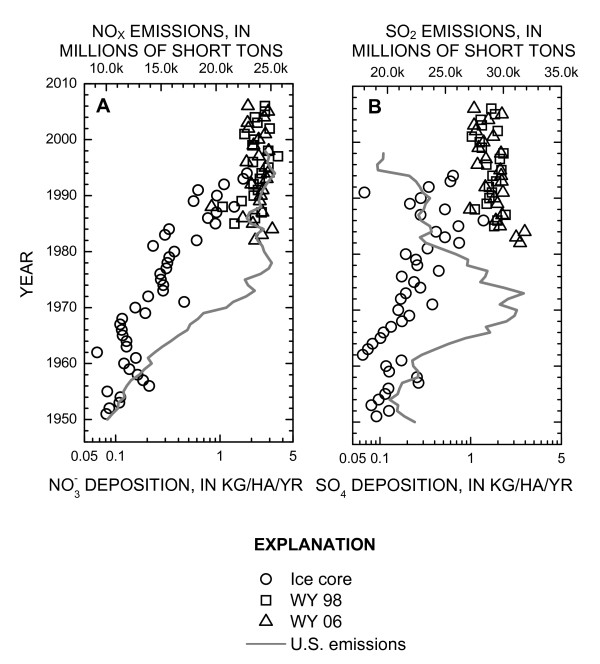
**Nitrate (A) and sulfate (B) annual wet deposition at NADP/NTN sites WY06 and WY98 and total deposition calculated from ice core DH-98-4 compared to total U.S. emissions of NO_x _(A) and SO_2 _(B) from 1950 to 1998 **[46].

In addition to the S and N isotope data, the ice-core samples were used to provide an archive of annual  and  deposition amounts that could be used to qualitatively extend the NADP/NTN records to the early-1950s. Dissolved (0.45 μm)  and  concentrations from the 1998 ice-core samples (site DH-98-4) were used in combination with the average ice-core density value of 0.90 g/cm^3 ^(n = 12) to calculate deposition masses in kilograms/hectare (kg/ha). The age of each ice-core sample from site DH-98-4 was determined from the modified age-depth profile developed for the 1991 ice core to account for the additional snow deposited between 1991 and 1998. Once an age was assigned to each ice-core sample, the  and  masses from the same year were summed to represent an annual deposition mass that could be directly compared to the NADP/NTN deposition data from sites WY06 and WY98 [52]. Annual  and  masses in kg/ha were calculated for snow deposited on UFG from 1951 through 1993 (figure 5).

Comparison of the  wet deposition data from the NADP/NTN sites to UFG data suggests that  deposition in the WRR may have been less prior to 1980 (figure 5). During the time period of overlapping  data there is some general agreement between UFG and NADP/NTN annual deposition masses (i.e. 1985, 1988, 1992, 1993, and 1994). Other years of overlapping data indicate substantially less annual  deposition from the ice-core record relative to NADP/NTN sites WY06 and WY98 (i.e. 1982, 1983, 1984, 1990, and 1991).

A number of reasons may explain the time periods when  deposition rates recorded in the UFG ice core were different than the measured deposition rates at the NADP/NTN sites. At 4,000 m ASL, the UFG ice-coring site is subject to wind erosion of previously deposited snow and the corresponding  deposition mass. An on-site snow depth sensor on UFG was used in combination with snow-density measurements to document significant snow removal by wind events after deposition [25]. Data collected on UFG during 1999-2000 indicated that the snow removal events were less during the spring, likely due to warmer air temperatures, resulting in higher-density snowfall. At an elevation of 4 km ASL, UFG is subject to free tropospheric and sometimes stratospheric air, increasing the likelihood of receiving atmospheric deposition from hemispheric sources relative to the lower elevation NADP/NTN sites. The NO_x _produced from the oxidation of nitrous oxide in the stratosphere should contain a δ^15^N of ~ 19 ‰ [35], which is significantly enriched relative to the δ^15^N () values observed in the UFG ice-core samples (figure 4).

Post-depositional elution of solutes from the snowpack deposited on UFG could also contribute to lower  masses relative to similar time periods at the NADP/NTN sites. However, chemical and isotopic analyses of ice from both cores indicate the low-resolution preservation of historical records of environmental change, including above-ground nuclear weapons testing, air temperature, volcanic eruptions, and the chemical quality of atmospheric deposition such as sub-μg/L concentrations of atmospheric mercury [20-27].

The deposition data from UFG (1951 through 1993) indicates an increasing trend in  deposition until approximately 1980 (figure 5). Beginning in 1980, both the NADP/NTN (1980 to 2006) sites and the UFG data indicate slightly decreasing to stable  deposition. During the time period of overlapping  deposition data from both UFG and NADP/NTN sites, there is only one year where similar deposition masses occur (1986). All remaining years of overlapping data indicate substantially less annual  deposition from the ice-core record relative to the NADP/NTN sites. Similar processes controlling  deposition also are likely affecting the annual  deposition rates on UFG relative to the NADP/NTN sites.

The annual  and  deposition data from UFG was compared with total U.S. emissions of NO_X _and SO_2 _from 1950 to 1998 (figure 5) as compiled by the U.S. Environmental Protection Agency. Although this comparison does not address the overall higher NO_X _and SO_2 _emissions in the eastern U.S. relative to Wyoming deposition rates, it does provide a historical perspective on the initiation of the NADP/NTN program relative to total emission amounts. The effect of sharply increasing NO_X _and SO_2 _emissions from 1950 to the mid-1970s occurred before the beginning of the NADP/NTN program; however, the UFG ice-core samples provide a record of the  and  deposition effects during this time of increasing emissions (figure 5). Although post-depositional elution processes may have decreased the total  and  masses, the trends in deposition from 1950 to the mid-1970s appear to reflect the sharply increasing U.S. emissions data during this same time period. The NADP/NTN program sites in Wyoming were initiated after the peak national SO_2 _emissions in 1973 and substantially smaller annual NO_X _emissions after 1978. The decrease in U.S. SO_2 _emissions after the early-1970s is not reflected by the UFG ice-core data (figure 5).

Given the observed increase in  and  deposition in the UFG ice-core samples since the mid-1940s, it is unclear why no distinctive temporal trend in the isotopic composition is observed during the same time period. This may suggest that most of the  and  deposition to the WRR since the mid-1940s has been dominated by anthropogenic inputs from a common source and any increases in deposition amounts contain similar isotopic signatures. Additional reasons for the lack of an isotopic trend could include: (1) limited period of record starting after the existence of anthropogenic inputs; (2) large sample composites for isotope samples covering multiple years of deposition that mask finer scale trends evident in the  and  deposition data; (3) varying deposition amounts from local, regional, and global sources; and (4) isotopic fractionation from postdepositional processes in the firn and ice. Additional studies are needed to assess the impact of postdepostional processes on the preservation of δ^34^S () and δ^15^N () values at the UFG coring site. Recent work at the Summit site, Greenland, found a potentially minimal influence of photolysis on the isotopic composition of  in firn and ice, in part due to high accumulation rates [18].

Isotopic analyses from other sections of the UFG ice core (including pre-industrial) would provide additional insight into the existence of isotopic trends. Unfortunately, the low concentration of  and  combined with an insufficient amount of ice-core samples representing atmospheric deposition prior to ~ 1945 does not allow for sufficient sample mass to support S and N isotopic analyses. Despite this short-coming, results from these ice-core data provide insight to the long-term wet deposition of  and  in pristine areas of the western United States that were previously not available.

## Summary

Glaciers in the WRR present a unique opportunity to couple short-term (1980 to present) NADP/NTN data trends in the chemistry of atmospheric deposition with longer-term data, including stable isotope data, preserved in glacial ice. The δ^34^S values in seven ice-core samples representing snowfall deposited from approximately 1946 to 1988 were similar, ranging from 5.1 to 8.1 ‰. The highest δ^34^S value in the UFG ice-core samples was associated with snow deposited primarily during 1980 and coincident with the eruption of Mt. St. Helens, Washington, during May 1980. The measured δ^34^S value of 8.1 ‰ in the ice core was in close agreement with the mean δ^34^S value of 8.3 ‰ of volcanic ash from the 1980 eruption. The remaining δ^34^S values from ice-core samples were substantially enriched relative to biogenic  sources and slightly depleted relative to δ^34^S values of water-soluble  from dry lake beds in arid regions of the southwestern United States. Coal used in power plants in close proximity to UFG was similar in δ^34^S isotopic composition suggesting that power plant emissions may be a dominant source of S deposition to the WRR.

The mean δ^15^N value in the UFG ice-core samples (-4.0 ‰, n = 7) was equal to the mean δ^15^N value of six archived precipitation samples collected during 2000 at NADP/NTN site WY98, suggesting similar  source(s). Ice-core δ^15^N values were substantially depleted relative to δ^15^N values of NO_X _from coal-fired powerplants, North American kerogen deposits, or simulated lightning and were substantially enriched relative to soil-released N. The ice-core samples were similar in isotopic composition to δ^15^N values measured from vehicle emissions. This similarity in isotopic composition is consistent with NO_X _emission data compiled for the U.S. during 1998 that indicates vehicle emissions as the dominant source of NO_X_.

Comparison of  and  wet deposition data from the NADP/NTN sites to UFG data suggests an increasing trend in annual deposition from 1950 to the installation of the NADP/NTN sites in western Wyoming during the early-1980s. Post-depositional elution processes may have decreased the total  and  masses preserved in UFG ice-core samples; however, the ice-core deposition data reflect the sharply increasing U.S. emissions from 1950 to the mid-1970s. Integration of the UFG ice-core data with the existing NADP/NTN data provides a longer-term, yet more qualitative, historical perspective on atmospheric deposition in the WRR.

## Competing interests

The authors declare that they have no competing interests.

## Authors' contributions

DN conceived the study, designed and coordinated study execution, assisted with sample processing, and drafted the manuscript. PS assisted in the design and execution of the study, assisted with sample processing, carried out the anion analyses, and assisted with the interpretation of the data. CJ carried out the isotopic analyses and provided assistance in drafting the manuscript. All authors read and approved the final manuscript.

## Supplementary Material

Additional file 1 **Supporting data**. Isotopic and anion data used in the study Click here for file
