# Supplementary material for: A 50-year record of NOx and SO2 sources in precipitation in the Northern Rocky Mountains, USA
Source: Geochem Trans. 2011 Mar 7;12:4. doi: 10.1186/1467-4866-12-4 (PMC3060858; doi:10.1186/1467-4866-12-4)
Supplement: Additional file 1 — Supporting data. Isotopic and anion data used in the study [file 1467-4866-12-4-S1.DOC]

**Table 1.** Nitrogen isotope values for NO_3_ determined from ice-core composite samples, Upper Fremont Glacier, Wyoming.

| Estimated age of deposition at mid-point of ice-core sample | δ^15^N, in permil |
| --- | --- |
| 2001* | 6 |
| 1987 | 5.5 |
| 1984 | 5.1 |
| 1980 | 8.1 |
| 1977 | 5.2 |
| 1972 | 6.7 |
| 1967 | 6.8 |
| 1946 | 5.8 |

*Snow sample collected from surface of Upper Fremont Glacier during summer 2001.

**Table 2.** Sulfur isotope values for SO_4_ determined from ice-core composite samples, Upper Fremont Glacier, Wyoming.

| Estimated age of deposition at mid-point of ice-core sample | δ^34^S, in permil |
| --- | --- |
| 1987 | -5.88 |
| 1984 | -3.74 |
| 1980 | -4.16 |
| 1977 | -3.19 |
| 1972 | -3.35 |
| 1967 | -3.74 |
| 1952 | -4.25 |

**Table 3.** Sample intervals, estimated time of deposition, nitrate concentration, and calculated nitrate mass in core DH-98-4, Upper Fremont Glacier, Wyoming.

[cm, centimeters; NO_3_, nitrate; mg/L, milligrams per liter; kg/ha, kilograms per hectare]

| Depth to top of sample interval, in cm | Depth to bottom of sample interval, in cm | Modeled age of ice core, in decimal years | NO_3_ concentration, in mg/L | Calculated NO_3_ mass, in kg/ha |
| --- | --- | --- | --- | --- |
| 623 | 630.36 | 1994.80969 | 0.34413 | 0.22802 |
| 630.36 | 637.72 | 1994.76192 | 0.38505 | 0.25514 |
| 637.72 | 645.08 | 1994.71408 | 0.22756 | 0.15078 |
| 645.08 | 652.44 | 1994.66615 | 0.14199 | 0.09409 |
| 652.44 | 659.8 | 1994.61815 | 0.13641 | 0.09039 |
| 659.8 | 667.16 | 1994.57007 | 0.11037 | 0.07313 |
| 667.16 | 674.52 | 1994.5219 | 0.11409 | 0.0756 |
| 674.52 | 681.88 | 1994.47366 | 0.13331 | 0.08833 |
| 681.88 | 689.24 | 1994.42533 | 0.15749 | 0.10436 |
| 689.24 | 696.6 | 1994.37693 | 0.15625 | 0.10354 |
| 696.6 | 703.96 | 1994.32844 | 0.15935 | 0.10559 |
| 703.96 | 711.32 | 1994.27988 | 0.12525 | 0.08299 |
| 711.32 | 718.68 | 1994.23123 | 0.11099 | 0.07354 |
| 718.68 | 726.04 | 1994.18251 | 0.11347 | 0.07519 |
| 726.04 | 733.4 | 1994.13371 | 0.09673 | 0.06409 |
| 733.4 | 740.76 | 1994.08482 | 0.11347 | 0.07519 |
| 740.76 | 748.12 | 1994.03586 | 0.10975 | 0.07272 |
| 748.12 | 755.48 | 1993.98681 | 0.07007 | 0.04643 |
| 755.48 | 762.84 | 1993.93769 | 0.06263 | 0.0415 |
| 762.84 | 770.2 | 1993.88848 | 0.07689 | 0.05095 |
| 770.2 | 777.56 | 1993.8392 | 0.10231 | 0.06779 |
| 777.56 | 784.92 | 1993.78983 | 0.08495 | 0.05629 |
| 784.92 | 792.28 | 1993.74038 | 0.07565 | 0.05012 |
| 792.28 | 799.64 | 1993.69086 | 0.09797 | 0.06491 |
| 799.64 | 807 | 1993.64125 | 0.11347 | 0.07519 |
| 807 | 816 | 1993.58603 | 0.19532 | 0.15826 |
| 816 | 830 | 1993.50822 | 0.12711 | 0.16021 |
| 830 | 841 | 1993.42342 | 0.15191 | 0.15044 |
| 841 | 852 | 1993.34861 | 0.16803 | 0.16641 |
| 852 | 863 | 1993.27362 | 0.18726 | 0.18544 |
| 863 | 874 | 1993.19846 | 0.16617 | 0.16456 |
| 874 | 885 | 1993.12311 | 0.14633 | 0.14492 |
| 885 | 896 | 1993.04758 | 0.09425 | 0.09334 |
| 896 | 907 | 1992.97188 | 0.09115 | 0.09026 |
| 907 | 918 | 1992.89599 | 0.09673 | 0.09579 |
| 918 | 929 | 1992.81993 | 0.11347 | 0.11237 |
| 929 | 940 | 1992.74369 | 0.06821 | 0.06755 |
| 940 | 951 | 1992.66727 | 0.08371 | 0.0829 |
| 951 | 962 | 1992.59067 | 0.10541 | 0.10439 |
| 962 | 973 | 1992.51389 | 0.08309 | 0.08228 |
| 973 | 981 | 1992.44744 | 0.07875 | 0.05672 |
| 981 | 992 | 1992.38085 | 0.10665 | 0.10562 |
| 992 | 1000 | 1992.31413 | 0.06449 | 0.04644 |
| 1000 | 1011 | 1992.24728 | 0.06325 | 0.06263 |
| 1011 | 1019.25 | 1992.17938 | 0.07751 | 0.05757 |
| 1019.25 | 1026.5 | 1992.12463 | 0.05146 | 0.03359 |
| 1026.5 | 1033.75 | 1992.07333 | 0.07751 | 0.05059 |
| 1033.75 | 1041 | 1992.02196 | 0.07007 | 0.04573 |
| 1041 | 1048.25 | 1991.97051 | 0.07007 | 0.04573 |
| 1048.25 | 1055.5 | 1991.91898 | 0.04712 | 0.03076 |
| 1055.5 | 1062.75 | 1991.86738 | 0.0527 | 0.0344 |
| 1062.75 | 1070 | 1991.81569 | 0.05022 | 0.03278 |
| 1070 | 1077.2 | 1991.76415 | 0.05456 | 0.03537 |
| 1077.2 | 1084.4 | 1991.71267 | 0.05952 | 0.03858 |
| 1084.4 | 1091.6 | 1991.66111 | 0.0558 | 0.03617 |
| 1091.6 | 1098.8 | 1991.60948 | 0.05828 | 0.03778 |
| 1098.8 | 1106 | 1991.55776 | 0.06325 | 0.041 |
| 1106 | 1113.2 | 1991.50598 | 0.04154 | 0.02693 |
| 1113.2 | 1120.4 | 1991.45411 | 0.03906 | 0.02532 |
| 1120.4 | 1127.6 | 1991.40217 | 0.04464 | 0.02894 |
| 1127.6 | 1134.8 | 1991.35016 | 0.031 | 0.0201 |
| 1134.8 | 1142 | 1991.29806 | 0.0372 | 0.02412 |
| 1142 | 1149.2 | 1991.24589 | 0.02666 | 0.01728 |
| 1149.2 | 1156.4 | 1991.19365 | 0.0434 | 0.02813 |
| 1156.4 | 1163.6 | 1991.14132 | 0.04774 | 0.03095 |
| 1163.6 | 1170.8 | 1991.08892 | 0.06697 | 0.04341 |
| 1170.8 | 1178 | 1991.03644 | 0.06077 | 0.03939 |
| 1178 | 1185.2 | 1990.98389 | 0.08061 | 0.05225 |
| 1185.2 | 1192.4 | 1990.93126 | 0.07441 | 0.04823 |
| 1192.4 | 1199.6 | 1990.87855 | 0.10169 | 0.06592 |
| 1199.6 | 1206.8 | 1990.82577 | 0.05766 | 0.03738 |
| 1206.8 | 1214 | 1990.77291 | 0.05828 | 0.03778 |
| 1214 | 1224 | 1990.70967 | 0.13455 | 0.12113 |
| 1224 | 1234 | 1990.636 | 0.13145 | 0.11834 |
| 1234 | 1244 | 1990.56218 | 0.11409 | 0.10271 |
| 1244 | 1254 | 1990.48822 | 0.06077 | 0.05471 |
| 1254 | 1264 | 1990.4141 | 0.04278 | 0.03852 |
| 1264 | 1274 | 1990.33984 | 0.05022 | 0.04522 |
| 1274 | 1284 | 1990.26543 | 0.08743 | 0.07871 |
| 1284 | 1294 | 1990.19087 | 0.0558 | 0.05024 |
| 1294 | 1304 | 1990.11617 | 0.0527 | 0.04745 |
| 1304 | 1312 | 1990.04881 | 0.04774 | 0.03439 |
| 1312 | 1322 | 1989.98133 | 0.04774 | 0.04298 |
| 1322 | 1332 | 1989.90621 | 0.04154 | 0.0374 |
| 1332 | 1340 | 1989.83847 | 0.03968 | 0.02858 |
| 1340 | 1348 | 1989.77817 | 0.03968 | 0.02858 |
| 1348 | 1356 | 1989.71776 | 0.0403 | 0.02903 |
| 1356 | 1364 | 1989.65727 | 0.03658 | 0.02635 |
| 1364 | 1372 | 1989.59667 | 0.04464 | 0.03215 |
| 1372 | 1380 | 1989.53599 | 0.05766 | 0.04153 |
| 1380 | 1388 | 1989.47521 | 0.04774 | 0.03439 |
| 1388 | 1396 | 1989.41433 | 0.0496 | 0.03573 |
| 1396 | 1404 | 1989.35336 | 0.0465 | 0.03349 |
| 1404 | 1412 | 1989.2923 | 0.0496 | 0.03573 |
| 1412 | 1420 | 1989.23114 | 0.0496 | 0.03573 |
| 1420 | 1430 | 1989.16222 | 0.04278 | 0.03852 |
| 1430 | 1437.8 | 1989.09395 | 0.06387 | 0.04485 |
| 1437.8 | 1445.6 | 1989.03402 | 0.0465 | 0.03266 |
| 1445.6 | 1453.4 | 1988.974 | 0.04588 | 0.03222 |
| 1453.4 | 1461.2 | 1988.9139 | 0.0527 | 0.03701 |
| 1461.2 | 1469 | 1988.8537 | 0.03906 | 0.02743 |
| 1469 | 1476.8 | 1988.79341 | 0.03844 | 0.027 |
| 1476.8 | 1484.6 | 1988.73303 | 0.05022 | 0.03527 |
| 1484.6 | 1492.4 | 1988.67256 | 0.0527 | 0.03701 |
| 1492.4 | 1500.2 | 1988.61201 | 0.06325 | 0.04441 |
| 1500.2 | 1508 | 1988.55136 | 0.09797 | 0.0688 |
| 1508 | 1516.4 | 1988.48829 | 0.14571 | 0.11019 |
| 1516.4 | 1524.2 | 1988.42511 | 0.18354 | 0.12888 |
| 1524.2 | 1532 | 1988.36419 | 0.21206 | 0.14891 |
| 1532 | 1539.8 | 1988.30318 | 0.18044 | 0.12671 |
| 1539.8 | 1547.6 | 1988.24207 | 0.19346 | 0.13585 |
| 1547.6 | 1555.4 | 1988.18088 | 0.20462 | 0.14369 |
| 1555.4 | 1563.2 | 1988.11959 | 0.21702 | 0.1524 |
| 1563.2 | 1571 | 1988.05822 | 0.16121 | 0.11321 |
| 1571 | 1578.8 | 1987.99676 | 0.08185 | 0.05747 |
| 1578.8 | 1586.6 | 1987.9352 | 0.16307 | 0.11451 |
| 1586.6 | 1594.4 | 1987.87356 | 0.18726 | 0.1315 |
| 1594.4 | 1602.2 | 1987.81183 | 0.16555 | 0.11626 |
| 1602.2 | 1610 | 1987.75 | 0.15253 | 0.10711 |
| 1610 | 1620 | 1987.67935 | 0.10169 | 0.09155 |
| 1620 | 1628.6 | 1987.6054 | 0.06325 | 0.04897 |
| 1628.6 | 1636.8 | 1987.53849 | 0.0527 | 0.03891 |
| 1636.8 | 1644.4 | 1987.47548 | 0.03906 | 0.02673 |
| 1644.4 | 1652 | 1987.41476 | 0.0248 | 0.01697 |
| 1652 | 1659.6 | 1987.35397 | 0.03162 | 0.02164 |
| 1659.6 | 1667.2 | 1987.29308 | 0.06015 | 0.04115 |
| 1667.2 | 1674.8 | 1987.23212 | 0.04836 | 0.03309 |
| 1674.8 | 1683 | 1987.16865 | 0.0527 | 0.03891 |
| 1683 | 1690.6 | 1987.10509 | 0.06263 | 0.04285 |
| 1698.2 | 1705.8 | 1986.98255 | 0.05952 | 0.04073 |
| 1705.8 | 1713.4 | 1986.92114 | 0.05146 | 0.03521 |
| 1713.4 | 1721 | 1986.85966 | 0.0434 | 0.0297 |
| 1721 | 1728.6 | 1986.79808 | 0.06759 | 0.04624 |
| 1728.6 | 1736.2 | 1986.73643 | 0.04464 | 0.03055 |
| 1736.2 | 1743.8 | 1986.67468 | 0.04278 | 0.02927 |
| 1743.8 | 1751.4 | 1986.61286 | 0.04588 | 0.03139 |
| 1751.4 | 1759 | 1986.55094 | 0.0465 | 0.03182 |
| 1759 | 1770.2 | 1986.47425 | 0.10293 | 0.10379 |
| 1770.2 | 1780.8 | 1986.38515 | 0.06387 | 0.06095 |
| 1780.8 | 1791.4 | 1986.29833 | 0.07627 | 0.07278 |
| 1791.4 | 1801.4 | 1986.21382 | 0.03224 | 0.02903 |
| 1801.4 | 1811.4 | 1986.13161 | 0.12277 | 0.11053 |
| 1811.4 | 1821.4 | 1986.04926 | 0.12525 | 0.11276 |
| 1821.4 | 1831.2 | 1985.96758 | 0.10293 | 0.09081 |
| 1831.2 | 1838.6 | 1985.89652 | 0.11347 | 0.07559 |
| 1838.6 | 1846 | 1985.83528 | 0.11657 | 0.07766 |
| 1846 | 1853.8 | 1985.7723 | 0.30755 | 0.21597 |
| 1853.8 | 1861.2 | 1985.70924 | 0.08371 | 0.05577 |
| 1861.2 | 1868.6 | 1985.64775 | 0.09053 | 0.06031 |
| 1868.6 | 1876 | 1985.58619 | 0.10603 | 0.07064 |
| 1876 | 1883.4 | 1985.52454 | 0.05704 | 0.038 |
| 1883.4 | 1890.8 | 1985.46281 | 0.02728 | 0.01818 |
| 1890.8 | 1898.8 | 1985.39849 | 0.04588 | 0.03305 |
| 1898.8 | 1906.2 | 1985.33409 | 0.04092 | 0.02726 |
| 1906.2 | 1913.6 | 1985.27211 | 0.04898 | 0.03263 |
| 1913.6 | 1921 | 1985.21005 | 0.03968 | 0.02644 |
| 1921 | 1928.4 | 1985.14791 | 0.03596 | 0.02396 |
| 1928.4 | 1935.8 | 1985.08569 | 0.03844 | 0.02561 |
| 1935.8 | 1943.6 | 1985.02171 | 0.05022 | 0.03527 |
| 1943.6 | 1951 | 1984.95764 | 0.03472 | 0.02313 |
| 1951 | 1958.4 | 1984.89517 | 0.0341 | 0.02272 |
| 1958.4 | 1965.8 | 1984.83262 | 0.03348 | 0.02231 |
| 1965.8 | 1973.2 | 1984.76999 | 0.0434 | 0.02892 |
| 1973.2 | 1980.6 | 1984.70728 | 0.03286 | 0.02189 |
| 1980.6 | 1988.45 | 1984.64253 | 0.04774 | 0.03374 |
| 1988.45 | 1995.9 | 1984.57753 | 0.03224 | 0.02163 |
| 1995.9 | 2003.35 | 1984.51415 | 0.03224 | 0.02163 |
| 2003.35 | 2010.8 | 1984.45068 | 0.03286 | 0.02204 |
| 2010.8 | 2018.25 | 1984.38714 | 0.02418 | 0.01622 |
| 2018.25 | 2025.7 | 1984.32351 | 0.02232 | 0.01497 |
| 2025.7 | 2034.6 | 1984.25791 | 0.02108 | 0.01689 |
| 2034.6 | 2042.05 | 1984.19214 | 0.02542 | 0.01705 |
| 2042.05 | 2049.5 | 1984.12826 | 0.02418 | 0.01622 |
| 2049.5 | 2056.95 | 1984.0643 | 0.01984 | 0.01331 |
| 2056.95 | 2064.4 | 1984.00025 | 0.01736 | 0.01164 |
| 2064.4 | 2071.85 | 1983.93612 | 0.02728 | 0.0183 |
| 2071.85 | 2079.3 | 1983.87192 | 0.02046 | 0.01372 |
| 2079.3 | 2086.75 | 1983.80762 | 0.02666 | 0.01788 |
| 2086.75 | 2094.2 | 1983.74325 | 0.03348 | 0.02246 |
| 2094.2 | 2101.65 | 1983.67879 | 0.03286 | 0.02204 |
| 2101.65 | 2109.1 | 1983.61426 | 0.03658 | 0.02454 |
| 2109.1 | 2116.55 | 1983.54964 | 0.02666 | 0.01788 |
| 2116.55 | 2124.45 | 1983.48302 | 0.0279 | 0.01984 |
| 2124.45 | 2131.9 | 1983.41624 | 0.03162 | 0.02121 |
| 2131.9 | 2139.35 | 1983.35137 | 0.04712 | 0.03161 |
| 2139.35 | 2146.8 | 1983.28641 | 0.03286 | 0.02204 |
| 2146.8 | 2154.7 | 1983.21946 | 0.03348 | 0.02381 |
| 2154.7 | 2162.15 | 1983.15233 | 0.02294 | 0.01539 |
| 2162.15 | 2169.6 | 1983.08712 | 0.01488 | 0.00998 |
| 2169.6 | 2177.05 | 1983.02184 | 0.02356 | 0.0158 |
| 2177.05 | 2184.5 | 1982.95647 | 0.02604 | 0.01747 |
| 2184.5 | 2194.75 | 1982.87871 | 0.01426 | 0.01316 |
| 2194.75 | 2205.67 | 1982.7856 | 0.18354 | 0.18044 |
| 2205.67 | 2215.81 | 1982.69276 | 0.05332 | 0.04868 |
| 2215.81 | 2223.28 | 1982.61497 | 0.03658 | 0.0246 |
| 2223.28 | 2230.75 | 1982.54891 | 0.0527 | 0.03544 |
| 2230.75 | 2238.22 | 1982.48278 | 0.05766 | 0.03878 |
| 2238.22 | 2245.69 | 1982.41656 | 0.06511 | 0.04378 |
| 2245.69 | 2253.16 | 1982.35026 | 0.06387 | 0.04295 |
| 2253.16 | 2260.63 | 1982.28387 | 0.05332 | 0.03586 |
| 2260.63 | 2268.1 | 1982.21741 | 0.02852 | 0.01918 |
| 2268.1 | 2275.57 | 1982.15086 | 0.02542 | 0.0171 |
| 2275.57 | 2283.04 | 1982.08423 | 0.02728 | 0.01835 |
| 2283.04 | 2290.98 | 1982.01546 | 0.08805 | 0.06294 |
| 2290.98 | 2298.45 | 1981.94651 | 0.04092 | 0.02752 |
| 2298.45 | 2305.92 | 1981.87963 | 0.03782 | 0.02544 |
| 2305.92 | 2313.39 | 1981.81266 | 0.02728 | 0.01835 |
| 2313.39 | 2320.86 | 1981.7457 | 0.02294 | 0.01543 |
| 2320.86 | 2328.33 | 1981.67857 | 0.02108 | 0.01418 |
| 2328.33 | 2335.8 | 1981.61136 | 0.0155 | 0.01042 |
| 2335.8 | 2343.27 | 1981.54406 | 0.01984 | 0.01334 |
| 2343.27 | 2350.74 | 1981.47669 | 0.01674 | 0.01126 |
| 2350.74 | 2358.21 | 1981.40922 | 0.01612 | 0.01084 |
| 2358.21 | 2365.68 | 1981.34168 | 0.01736 | 0.01168 |
| 2365.68 | 2373.15 | 1981.27406 | 0.02356 | 0.01585 |
| 2373.15 | 2381.09 | 1981.20417 | 0.0186 | 0.0133 |
| 2381.09 | 2388.56 | 1981.13429 | 0.02542 | 0.0171 |
| 2388.56 | 2396.03 | 1981.06641 | 0.03472 | 0.02335 |
| 2396.03 | 2403.6 | 1980.99799 | 0.04216 | 0.02874 |
| 2403.6 | 2410.63 | 1980.9315 | 0.02604 | 0.01648 |
| 2410.63 | 2417.66 | 1980.86739 | 0.031 | 0.01962 |
| 2417.66 | 2424.69 | 1980.80321 | 0.03658 | 0.02315 |
| 2424.69 | 2431.72 | 1980.73895 | 0.01922 | 0.01217 |
| 2431.72 | 2438.75 | 1980.67462 | 0.02294 | 0.01452 |
| 2438.75 | 2445.78 | 1980.61022 | 0.05208 | 0.03296 |
| 2445.78 | 2452.81 | 1980.54575 | 0.04774 | 0.03022 |
| 2452.81 | 2459.84 | 1980.4812 | 0.03906 | 0.02472 |
| 2459.84 | 2466.9 | 1980.41639 | 0.05456 | 0.03468 |
| 2466.9 | 2473.93 | 1980.35161 | 0.0434 | 0.02747 |
| 2473.93 | 2480.96 | 1980.28684 | 0.0248 | 0.0157 |
| 2480.96 | 2487.99 | 1980.222 | 0.03658 | 0.02315 |
| 2487.99 | 2495.02 | 1980.15709 | 0.03782 | 0.02394 |
| 2495.02 | 2502.05 | 1980.09201 | 0.03286 | 0.0208 |
| 2502.05 | 2508.58 | 1980.02926 | 0.02976 | 0.0175 |
| 2508.58 | 2516.11 | 1979.96413 | 0.02852 | 0.01934 |
| 2516.11 | 2523.14 | 1979.89661 | 0.02852 | 0.01805 |
| 2523.14 | 2530.17 | 1979.83133 | 0.02852 | 0.01805 |
| 2530.17 | 2537.2 | 1979.76598 | 0.03348 | 0.02119 |
| 2537.2 | 2544.23 | 1979.70055 | 0.02852 | 0.01805 |
| 2544.23 | 2551.26 | 1979.63506 | 0.02542 | 0.01609 |
| 2551.26 | 2558.32 | 1979.56939 | 0.02418 | 0.01537 |
| 2558.32 | 2565.35 | 1979.50356 | 0.03596 | 0.02276 |
| 2565.35 | 2572.38 | 1979.43784 | 0.03782 | 0.02394 |
| 2572.38 | 2579.41 | 1979.37205 | 0.06573 | 0.0416 |
| 2579.41 | 2586.44 | 1979.30619 | 0.0527 | 0.03336 |
| 2586.44 | 2593.47 | 1979.24025 | 0.03844 | 0.02433 |
| 2593.47 | 2600.55 | 1979.17406 | 0.03224 | 0.02055 |
| 2600.55 | 2607.6 | 1979.1076 | 0.02914 | 0.0185 |
| 2607.6 | 2614.65 | 1979.04125 | 0.02418 | 0.01535 |
| 2614.65 | 2621.7 | 1978.97484 | 0.031 | 0.01968 |
| 2621.7 | 2628.75 | 1978.90834 | 0.03224 | 0.02046 |
| 2628.75 | 2635.8 | 1978.84178 | 0.03658 | 0.02322 |
| 2635.8 | 2642.85 | 1978.77514 | 0.02852 | 0.0181 |
| 2642.85 | 2649.9 | 1978.70843 | 0.02852 | 0.0181 |
| 2649.9 | 2657.12 | 1978.64089 | 0.02976 | 0.01935 |
| 2657.12 | 2664.14 | 1978.57336 | 0.02294 | 0.0145 |
| 2664.14 | 2671.16 | 1978.50671 | 0.03286 | 0.02077 |
| 2671.16 | 2678.18 | 1978.43999 | 0.05704 | 0.03605 |
| 2678.18 | 2685.2 | 1978.3732 | 0.03162 | 0.01999 |
| 2685.2 | 2692.22 | 1978.30633 | 0.03224 | 0.02038 |
| 2692.22 | 2699.24 | 1978.23939 | 0.02852 | 0.01803 |
| 2699.24 | 2706.26 | 1978.17238 | 0.04154 | 0.02626 |
| 2706.26 | 2713.28 | 1978.10529 | 0.02852 | 0.01803 |
| 2713.28 | 2720.3 | 1978.03813 | 0.03224 | 0.02038 |
| 2720.3 | 2727.34 | 1977.97081 | 0.03038 | 0.01926 |
| 2727.34 | 2734.36 | 1977.90341 | 0.04278 | 0.02704 |
| 2734.36 | 2741.38 | 1977.83603 | 0.05766 | 0.03644 |
| 2741.38 | 2748.4 | 1977.76858 | 0.03596 | 0.02273 |
| 2748.4 | 2755.42 | 1977.70106 | 0.0372 | 0.02351 |
| 2755.42 | 2762.44 | 1977.63346 | 0.02914 | 0.01842 |
| 2762.44 | 2769.46 | 1977.56579 | 0.03038 | 0.0192 |
| 2769.46 | 2776.48 | 1977.49805 | 0.0248 | 0.01567 |
| 2776.48 | 2783.57 | 1977.42985 | 0.03348 | 0.02137 |
| 2783.57 | 2790.7 | 1977.36109 | 0.02542 | 0.01632 |
| 2790.7 | 2797.83 | 1977.29206 | 0.02542 | 0.01632 |
| 2797.83 | 2804.96 | 1977.22296 | 0.02852 | 0.01831 |
| 2804.96 | 2812.09 | 1977.15378 | 0.0279 | 0.01791 |
| 2812.09 | 2819.35 | 1977.08395 | 0.02728 | 0.01783 |
| 2819.35 | 2826.48 | 1977.01394 | 0.02418 | 0.01552 |
| 2826.48 | 2833.61 | 1976.94454 | 0.02914 | 0.01871 |
| 2833.61 | 2840.74 | 1976.87506 | 0.03038 | 0.0195 |
| 2840.74 | 2847.87 | 1976.8055 | 0.04216 | 0.02706 |
| 2847.87 | 2854.86 | 1976.73656 | 0.02666 | 0.01678 |
| 2854.86 | 2861.82 | 1976.66842 | 0.02294 | 0.01438 |
| 2861.82 | 2868.78 | 1976.60031 | 0.02356 | 0.01476 |
| 2868.78 | 2875.74 | 1976.53212 | 0.01922 | 0.01204 |
| 2875.74 | 2882.7 | 1976.46387 | 0.0248 | 0.01554 |
| 2882.7 | 2889.66 | 1976.39554 | 0.0217 | 0.0136 |
| 2889.66 | 2896.62 | 1976.32714 | 0.02294 | 0.01438 |
| 2896.62 | 2903.58 | 1976.25867 | 0.0558 | 0.03497 |
| 2903.58 | 2910.54 | 1976.19013 | 0.03286 | 0.02059 |
| 2910.54 | 2917.5 | 1976.12151 | 0.0372 | 0.02331 |
| 2917.5 | 2924.46 | 1976.05283 | 0.03596 | 0.02253 |
| 2924.46 | 2931.82 | 1975.98209 | 0.01922 | 0.01274 |
| 2931.82 | 2938.78 | 1975.91128 | 0.02108 | 0.01321 |
| 2938.78 | 2945.74 | 1975.84238 | 0.01922 | 0.01204 |
| 2945.74 | 2952.7 | 1975.7734 | 0.02728 | 0.0171 |
| 2952.7 | 2959.66 | 1975.70436 | 0.02108 | 0.01321 |
| 2959.66 | 2966.62 | 1975.63524 | 0.0248 | 0.01554 |
| 2966.62 | 2973.58 | 1975.56605 | 0.02356 | 0.01476 |
| 2973.58 | 2980.54 | 1975.49678 | 0.05022 | 0.03147 |
| 2980.54 | 2988.1 | 1975.42446 | 0.0279 | 0.01899 |
| 2988.1 | 2995.55 | 1975.34956 | 0.03968 | 0.02662 |
| 2995.55 | 3003 | 1975.27518 | 0.04092 | 0.02745 |
| 3003 | 3010.45 | 1975.20072 | 0.04464 | 0.02994 |
| 3010.45 | 3017.9 | 1975.12618 | 0.03038 | 0.02038 |
| 3017.9 | 3025.35 | 1975.05155 | 0.02604 | 0.01747 |
| 3025.35 | 3032.8 | 1974.97684 | 0.03472 | 0.02329 |
| 3032.8 | 3040.25 | 1974.90205 | 0.031 | 0.02079 |
| 3040.25 | 3047.7 | 1974.82718 | 0.0434 | 0.02911 |
| 3047.7 | 3055.15 | 1974.75222 | 0.02666 | 0.01788 |
| 3055.15 | 3062.6 | 1974.67719 | 0.031 | 0.02079 |
| 3062.6 | 3070.05 | 1974.60207 | 0.02976 | 0.01996 |
| 3070.05 | 3077.95 | 1974.52465 | 0.02542 | 0.01808 |
| 3077.95 | 3085.85 | 1974.44481 | 0.02542 | 0.01808 |
| 3085.85 | 3093.3 | 1974.36711 | 0.031 | 0.02079 |
| 3093.3 | 3100.75 | 1974.29165 | 0.04836 | 0.03244 |
| 3100.75 | 3108.2 | 1974.21611 | 0.03286 | 0.02204 |
| 3108.2 | 3116.1 | 1974.13826 | 0.02852 | 0.02029 |
| 3116.1 | 3123.55 | 1974.06022 | 0.03348 | 0.02246 |
| 3123.55 | 3131.33 | 1973.9828 | 6.20052E-4 | 4.3429E-4 |
| 3131.33 | 3138.62 | 1973.90601 | 0.0248 | 0.01628 |
| 3138.62 | 3145.91 | 1973.83168 | 0.02976 | 0.01953 |
| 3145.91 | 3153.2 | 1973.75728 | 0.02914 | 0.01913 |
| 3153.2 | 3160.49 | 1973.6828 | 0.02232 | 0.01465 |
| 3160.49 | 3167.78 | 1973.60824 | 0.02852 | 0.01872 |
| 3167.78 | 3175.07 | 1973.5336 | 0.03596 | 0.0236 |
| 3175.07 | 3182.36 | 1973.45888 | 0.02604 | 0.01709 |
| 3182.36 | 3189.65 | 1973.38409 | 0.02914 | 0.01913 |
| 3189.65 | 3196.94 | 1973.30921 | 0.03968 | 0.02604 |
| 3196.94 | 3204.23 | 1973.23426 | 0.06635 | 0.04354 |
| 3204.23 | 3211.52 | 1973.15923 | 0.04526 | 0.02971 |
| 3211.52 | 3218.81 | 1973.08412 | 0.03224 | 0.02116 |
| 3218.81 | 3226.1 | 1973.00893 | 0.02418 | 0.01587 |
| 3226.1 | 3233.39 | 1972.93366 | 0.02914 | 0.01913 |
| 3233.39 | 3240.68 | 1972.85832 | 0.02108 | 0.01384 |
| 3240.68 | 3247.97 | 1972.78289 | 0.02604 | 0.01709 |
| 3247.97 | 3255.55 | 1972.70594 | 0.02046 | 0.01396 |
| 3255.55 | 3262.84 | 1972.6288 | 0.02418 | 0.01587 |
| 3262.84 | 3270.13 | 1972.55314 | 0.02232 | 0.01465 |
| 3270.13 | 3277.42 | 1972.47739 | 0.0248 | 0.01628 |
| 3277.42 | 3284.71 | 1972.40157 | 0.0217 | 0.01424 |
| 3284.71 | 3292.64 | 1972.32234 | 0.01736 | 0.01239 |
| 3292.64 | 3300.26 | 1972.24136 | 0.02852 | 0.01957 |
| 3300.26 | 3307.88 | 1972.16185 | 0.02294 | 0.01574 |
| 3307.88 | 3315.5 | 1972.08225 | 0.02728 | 0.01872 |
| 3315.5 | 3323.12 | 1972.00257 | 0.01922 | 0.01319 |
| 3323.12 | 3330.74 | 1971.9228 | 0.01674 | 0.01148 |
| 3330.74 | 3338.36 | 1971.84295 | 0.01612 | 0.01106 |
| 3338.36 | 3345.98 | 1971.76301 | 0.01736 | 0.01191 |
| 3345.98 | 3353.6 | 1971.68299 | 0.0186 | 0.01276 |
| 3353.6 | 3361.22 | 1971.60288 | 0.02294 | 0.01574 |
| 3361.22 | 3368.84 | 1971.52268 | 0.03348 | 0.02297 |
| 3368.84 | 3376.46 | 1971.4424 | 0.01488 | 0.01021 |
| 3376.46 | 3384.08 | 1971.36203 | 0.01364 | 0.00936 |
| 3384.08 | 3391.7 | 1971.28158 | 0.0186 | 0.01276 |
| 3391.7 | 3399.32 | 1971.20104 | 0.0155 | 0.01063 |
| 3399.32 | 3406.94 | 1971.12041 | 0.44334 | 0.30414 |
| 3406.94 | 3414.56 | 1971.0397 | 0.03162 | 0.02169 |
| 3414.56 | 3422.18 | 1970.95891 | 0.02294 | 0.01574 |
| 3422.18 | 3429.8 | 1970.87802 | 0.03038 | 0.02084 |
| 3429.8 | 3438.04 | 1970.79376 | 0.02356 | 0.01748 |
| 3438.04 | 3445.66 | 1970.7094 | 0.0186 | 0.01276 |
| 3445.66 | 3453.28 | 1970.62826 | 0.02542 | 0.01744 |
| 3453.28 | 3460.9 | 1970.54702 | 0.01612 | 0.01106 |
| 3460.9 | 3469.14 | 1970.46239 | 0.01612 | 0.01196 |
| 3469.14 | 3476.76 | 1970.37767 | 0.0093 | 0.00638 |
| 3476.76 | 3484.38 | 1970.29618 | 0.01922 | 0.01319 |
| 3484.38 | 3492.56 | 1970.21159 | 0.01302 | 0.00959 |
| 3492.56 | 3499.92 | 1970.12831 | 0.01426 | 0.00945 |
| 3499.92 | 3507.28 | 1970.04934 | 0.01302 | 0.00863 |
| 3507.28 | 3514.64 | 1969.9703 | 0.01612 | 0.01068 |
| 3514.64 | 3522.36 | 1969.88923 | 0.01426 | 0.00991 |
| 3522.36 | 3529.72 | 1969.80808 | 0.0062 | 0.00411 |
| 3529.72 | 3537.08 | 1969.72879 | 0.01488 | 0.00986 |
| 3537.08 | 3544.44 | 1969.64942 | 0.02666 | 0.01767 |
| 3544.44 | 3551.8 | 1969.56996 | 0.01798 | 0.01191 |
| 3551.8 | 3559.16 | 1969.49043 | 0.02666 | 0.01767 |
| 3559.16 | 3566.52 | 1969.41082 | 0.02604 | 0.01726 |
| 3566.52 | 3573.88 | 1969.33113 | 0.0217 | 0.01438 |
| 3573.88 | 3581.24 | 1969.25135 | 0.0279 | 0.01849 |
| 3581.24 | 3588.6 | 1969.1715 | 0.02542 | 0.01684 |
| 3588.6 | 3595.96 | 1969.09157 | 0.02356 | 0.01561 |
| 3595.96 | 3603.32 | 1969.01155 | 0.04216 | 0.02794 |
| 3603.32 | 3611.04 | 1968.9295 | 0.02542 | 0.01767 |
| 3611.04 | 3618.4 | 1968.84736 | 0.01364 | 0.00904 |
| 3618.4 | 3625.76 | 1968.76711 | 0.01426 | 0.00945 |
| 3625.76 | 3633.12 | 1968.68677 | 0.01364 | 0.00904 |
| 3633.12 | 3640.48 | 1968.60635 | 0.01426 | 0.00945 |
| 3640.48 | 3647.84 | 1968.52585 | 0.0124 | 0.00822 |
| 3647.84 | 3655.2 | 1968.44528 | 0.00744 | 0.00493 |
| 3655.2 | 3662.56 | 1968.36462 | 0.0124 | 0.00822 |
| 3662.56 | 3670.28 | 1968.28191 | 0.01426 | 0.00991 |
| 3670.28 | 3677.64 | 1968.19911 | 0.01364 | 0.00904 |
| 3677.64 | 3686.72 | 1968.10875 | 0.01488 | 0.01216 |
| 3686.72 | 3694.36 | 1968.01675 | 0.0124 | 0.00853 |
| 3694.36 | 3703.64 | 1967.92354 | 0.01674 | 0.01399 |
| 3703.64 | 3711.28 | 1967.83023 | 0.01736 | 0.01194 |
| 3711.28 | 3718.92 | 1967.74587 | 0.01426 | 0.00981 |
| 3718.92 | 3726.56 | 1967.66142 | 0.01488 | 0.01024 |
| 3726.56 | 3734.2 | 1967.57689 | 0.01488 | 0.01024 |
| 3734.2 | 3741.84 | 1967.49227 | 0.02666 | 0.01834 |
| 3741.84 | 3749.48 | 1967.40757 | 0.01364 | 0.00938 |
| 3749.48 | 3757.12 | 1967.32278 | 0.01488 | 0.01024 |
| 3757.12 | 3764.76 | 1967.2379 | 6.20052E-4 | 4.2648E-4 |
| 3764.76 | 3772.4 | 1967.15294 | 0.01054 | 0.00725 |
| 3772.4 | 3780.04 | 1967.06789 | 0.0124 | 0.00853 |
| 3780.04 | 3787.68 | 1966.98275 | 0.0186 | 0.01279 |
| 3787.68 | 3795.32 | 1966.89753 | 0.01054 | 0.00725 |
| 3795.32 | 3802.96 | 1966.81222 | 0.0155 | 0.01066 |
| 3802.96 | 3810.6 | 1966.72683 | 0.01302 | 0.00896 |
| 3810.6 | 3818.24 | 1966.64135 | 0.01054 | 0.00725 |
| 3818.24 | 3825.88 | 1966.55578 | 0.01054 | 0.00725 |
| 3825.88 | 3833.52 | 1966.47013 | 0.01426 | 0.00981 |
| 3833.52 | 3841.16 | 1966.38439 | 0.0155 | 0.01066 |
| 3841.16 | 3848.8 | 1966.29856 | 0.00806 | 0.00554 |
| 3848.8 | 3856.44 | 1966.21265 | 0.01302 | 0.00896 |
| 3856.44 | 3864.08 | 1966.12665 | 0.01984 | 0.01365 |
| 3864.08 | 3871.72 | 1966.04057 | 0.01674 | 0.01152 |
| 3871.72 | 3883 | 1965.93385 | 0.0186 | 0.01889 |
| 3883 | 3890.32 | 1965.82882 | 0.00992 | 0.00654 |
| 3890.32 | 3897.63 | 1965.74617 | 0.01116 | 0.00735 |
| 3897.63 | 3904.94 | 1965.66344 | 0.00992 | 0.00653 |
| 3904.94 | 3912.25 | 1965.58063 | 0.01054 | 0.00694 |
| 3912.25 | 3919.56 | 1965.49775 | 0.01302 | 0.00857 |
| 3919.56 | 3926.87 | 1965.41478 | 0.0248 | 0.01632 |
| 3926.87 | 3934.18 | 1965.33173 | 0.01426 | 0.00939 |
| 3934.18 | 3941.49 | 1965.24861 | 0.01984 | 0.01306 |
| 3941.49 | 3948.8 | 1965.16541 | 0.02046 | 0.01347 |
| 3948.8 | 3956.11 | 1965.08213 | 0.01426 | 0.00939 |
| 3956.11 | 3963.73 | 1964.99694 | 6.20052E-4 | 4.2537E-4 |
| 3963.73 | 3971.04 | 1964.91179 | 0.0155 | 0.0102 |
| 3971.04 | 3978.35 | 1964.82826 | 0.01302 | 0.00857 |
| 3978.35 | 3985.66 | 1964.74466 | 0.0155 | 0.0102 |
| 3985.66 | 3992.97 | 1964.66098 | 0.01302 | 0.00857 |
| 3992.97 | 4000.28 | 1964.57722 | 0.01798 | 0.01183 |
| 4000.28 | 4007.59 | 1964.49339 | 0.02232 | 0.01469 |
| 4007.59 | 4014.9 | 1964.40947 | 0.01674 | 0.01102 |
| 4014.9 | 4022.21 | 1964.32547 | 0.0124 | 0.00816 |
| 4022.21 | 4029.52 | 1964.2414 | 0.02666 | 0.01755 |
| 4029.52 | 4036.83 | 1964.15725 | 0.02728 | 0.01795 |
| 4036.83 | 4044.14 | 1964.07301 | 0.01178 | 0.00775 |
| 4044.14 | 4051.76 | 1963.98685 | 0.01984 | 0.01361 |
| 4051.76 | 4059.07 | 1963.90073 | 0.01736 | 0.01143 |
| 4059.07 | 4066.38 | 1963.81626 | 0.02108 | 0.01387 |
| 4066.38 | 4073.69 | 1963.7317 | 0.01364 | 0.00898 |
| 4073.69 | 4081.3 | 1963.64534 | 0.02108 | 0.01444 |
| 4081.3 | 4088.7 | 1963.55831 | 0.01922 | 0.01281 |
| 4088.7 | 4096.1 | 1963.47247 | 0.01798 | 0.01198 |
| 4096.1 | 4103.5 | 1963.38655 | 0.01674 | 0.01115 |
| 4103.5 | 4110.9 | 1963.30055 | 0.00868 | 0.00578 |
| 4110.9 | 4118.3 | 1963.21447 | 0.0124 | 0.00826 |
| 4118.3 | 4125.7 | 1963.12831 | 0.01178 | 0.00785 |
| 4125.7 | 4133.1 | 1963.04207 | 0.00992 | 0.00661 |
| 4133.1 | 4140.9 | 1962.95341 | 0.0093 | 0.00653 |
| 4140.9 | 4148.3 | 1962.86467 | 0.01116 | 0.00744 |
| 4148.3 | 4155.7 | 1962.77818 | 0.01054 | 0.00702 |
| 4155.7 | 4163.1 | 1962.69161 | 0.01426 | 0.0095 |
| 4163.1 | 4170.5 | 1962.60496 | 0.0093 | 0.0062 |
| 4170.5 | 4177.9 | 1962.51823 | 0.00992 | 0.00661 |
| 4177.9 | 4185.3 | 1962.43141 | 6.20052E-4 | 4.1309E-4 |
| 4185.3 | 4192.7 | 1962.34452 | 0.01178 | 0.00785 |
| 4192.7 | 4200.1 | 1962.25755 | 0.00868 | 0.00578 |
| 4200.1 | 4207.5 | 1962.17049 | 0.00496 | 0.0033 |
| 4207.5 | 4214.9 | 1962.08335 | 0.00868 | 0.00578 |
| 4214.9 | 4222.3 | 1961.99614 | 0.0124 | 0.00826 |
| 4222.3 | 4230.1 | 1961.90648 | 0.01488 | 0.01045 |
| 4230.1 | 4237.5 | 1961.81673 | 0.01488 | 0.00991 |
| 4237.5 | 4244.9 | 1961.72927 | 0.03286 | 0.02189 |
| 4244.9 | 4252.3 | 1961.64172 | 0.0248 | 0.01652 |
| 4252.3 | 4259.7 | 1961.5541 | 0.03348 | 0.02231 |
| 4259.7 | 4267.1 | 1961.46639 | 0.01736 | 0.01157 |
| 4267.1 | 4274.92 | 1961.37611 | 0.0186 | 0.0131 |
| 4274.92 | 4282.28 | 1961.28598 | 0.02108 | 0.01397 |
| 4282.28 | 4289.64 | 1961.1985 | 0.01116 | 0.0074 |
| 4289.64 | 4297 | 1961.11094 | 0.01674 | 0.01109 |
| 4297 | 4304.36 | 1961.0233 | 0.0155 | 0.01027 |
| 4304.36 | 4311.72 | 1960.93558 | 0.01302 | 0.00863 |
| 4311.72 | 4319.44 | 1960.84564 | 0.03224 | 0.02241 |
| 4319.44 | 4326.8 | 1960.7556 | 0.01488 | 0.00986 |
| 4326.8 | 4334.16 | 1960.66764 | 0.01674 | 0.01109 |
| 4334.16 | 4341.52 | 1960.5796 | 0.01488 | 0.00986 |
| 4341.52 | 4348.88 | 1960.49147 | 0.02108 | 0.01397 |
| 4348.88 | 4356.24 | 1960.40327 | 0.01426 | 0.00945 |
| 4356.24 | 4363.6 | 1960.31499 | 0.0186 | 0.01233 |
| 4363.6 | 4370.96 | 1960.22662 | 0.01426 | 0.00945 |
| 4370.96 | 4378.32 | 1960.13818 | 0.01178 | 0.00781 |
| 4378.32 | 4385.68 | 1960.04966 | 0.00868 | 0.00575 |
| 4385.68 | 4393.04 | 1959.96105 | 0.0155 | 0.01027 |
| 4393.04 | 4400.4 | 1959.87237 | 0.0186 | 0.01233 |
| 4400.4 | 4408.12 | 1959.78143 | 0.01178 | 0.00819 |
| 4408.12 | 4415.48 | 1959.69041 | 0.0155 | 0.01027 |
| 4415.48 | 4422.84 | 1959.60148 | 0.02232 | 0.01479 |
| 4422.84 | 4430.2 | 1959.51248 | 0.02604 | 0.01726 |
| 4430.2 | 4437.56 | 1959.42339 | 0.01798 | 0.01191 |
| 4437.56 | 4444.92 | 1959.33422 | 0.0186 | 0.01233 |
| 4444.92 | 4452.28 | 1959.24497 | 0.01612 | 0.01068 |
| 4452.28 | 4459.64 | 1959.15564 | 0.01984 | 0.01315 |
| 4459.64 | 4467 | 1959.06624 | 0.02108 | 0.01397 |
| 4467 | 4477.16 | 1958.95972 | 0.02418 | 0.02212 |
| 4477.16 | 4487.44 | 1958.8353 | 0.01798 | 0.01664 |
| 4487.44 | 4495 | 1958.72658 | 0.01364 | 0.00928 |
| 4495 | 4502.28 | 1958.63606 | 0.01426 | 0.00935 |
| 4502.28 | 4512.84 | 1958.52713 | 0.02976 | 0.0283 |
| 4512.84 | 4520.12 | 1958.41808 | 0.02666 | 0.01747 |
| 4520.12 | 4527.4 | 1958.32899 | 0.01922 | 0.0126 |
| 4527.4 | 4534.68 | 1958.23982 | 0.02356 | 0.01544 |
| 4534.68 | 4541.96 | 1958.15058 | 0.0248 | 0.01626 |
| 4541.96 | 4549.24 | 1958.06126 | 0.02232 | 0.01463 |
| 4549.24 | 4556.52 | 1957.97186 | 0.01984 | 0.013 |
| 4556.52 | 4563.8 | 1957.88238 | 0.01922 | 0.0126 |
| 4563.8 | 4571.08 | 1957.79282 | 0.02232 | 0.01463 |
| 4571.08 | 4578.36 | 1957.70319 | 0.0217 | 0.01422 |
| 4578.36 | 4585.92 | 1957.61175 | 0.01612 | 0.01097 |
| 4585.92 | 4593.2 | 1957.52023 | 0.02976 | 0.01951 |
| 4593.2 | 4600.48 | 1957.43035 | 0.0248 | 0.01626 |
| 4600.48 | 4607.76 | 1957.3404 | 0.02852 | 0.01869 |
| 4607.76 | 4615.04 | 1957.25037 | 0.03906 | 0.0256 |
| 4615.04 | 4622.32 | 1957.16026 | 0.02728 | 0.01788 |
| 4622.32 | 4629.6 | 1957.07007 | 0.02976 | 0.01951 |
| 4629.6 | 4636.88 | 1956.97981 | 0.03038 | 0.01991 |
| 4636.88 | 4644.16 | 1956.88947 | 0.031 | 0.02032 |
| 4644.16 | 4651.44 | 1956.79904 | 0.02542 | 0.01666 |
| 4651.44 | 4658.72 | 1956.70854 | 0.0279 | 0.01829 |
| 4658.72 | 4666.24 | 1956.61647 | 0.0527 | 0.03568 |
| 4666.24 | 4673.66 | 1956.52345 | 0.02914 | 0.01947 |
| 4673.66 | 4681.08 | 1956.43096 | 0.02232 | 0.01491 |
| 4681.08 | 4688.5 | 1956.3384 | 0.02108 | 0.01408 |
| 4688.5 | 4695.92 | 1956.24575 | 0.02914 | 0.01947 |
| 4695.92 | 4703.34 | 1956.15302 | 0.0279 | 0.01864 |
| 4703.34 | 4710.76 | 1956.06021 | 0.01798 | 0.01201 |
| 4710.76 | 4718.18 | 1955.96732 | 6.20052E-4 | 4.1420E-4 |
| 4718.18 | 4725.6 | 1955.87435 | 0.01922 | 0.01284 |
| 4725.6 | 4733.02 | 1955.78129 | 0.0155 | 0.01036 |
| 4733.02 | 4740.44 | 1955.68816 | 0.0124 | 0.00828 |
| 4740.44 | 4747.86 | 1955.59494 | 0.0124 | 0.00828 |
| 4747.86 | 4755.7 | 1955.499 | 0.00992 | 0.007 |
| 4755.7 | 4763.12 | 1955.40298 | 0.00744 | 0.00497 |
| 4763.12 | 4770.54 | 1955.30951 | 0.01178 | 0.00787 |
| 4770.54 | 4777.96 | 1955.21596 | 0.01116 | 0.00746 |
| 4777.96 | 4785.38 | 1955.12233 | 0.01302 | 0.0087 |
| 4785.38 | 4792.8 | 1955.02863 | 0.01054 | 0.00704 |
| 4792.8 | 4800.22 | 1954.93483 | 0.0155 | 0.01036 |
| 4800.22 | 4807.64 | 1954.84096 | 0.01798 | 0.01201 |
| 4807.64 | 4815.06 | 1954.74701 | 0.01364 | 0.00911 |
| 4815.06 | 4822.48 | 1954.65297 | 0.01612 | 0.01077 |
| 4822.48 | 4829.9 | 1954.55886 | 0.03038 | 0.0203 |
| 4829.9 | 4837.32 | 1954.46466 | 0.01488 | 0.00994 |
| 4837.32 | 4845.16 | 1954.36771 | 0.01736 | 0.01225 |
| 4845.16 | 4852.58 | 1954.27068 | 6.20052E-4 | 4.1420E-4 |
| 4852.58 | 4860 | 1954.17623 | 0.01302 | 0.0087 |
| 4860 | 4867.42 | 1954.0817 | 0.02542 | 0.01698 |
| 4867.42 | 4874.78 | 1953.98748 | 0.01736 | 0.0115 |
| 4874.78 | 4882.14 | 1953.89355 | 0.01178 | 0.00781 |
| 4882.14 | 4889.5 | 1953.79955 | 0.01364 | 0.00904 |
| 4889.5 | 4896.86 | 1953.70546 | 0.01054 | 0.00698 |
| 4896.86 | 4904.22 | 1953.6113 | 0.01426 | 0.00945 |
| 4904.22 | 4911.58 | 1953.51705 | 0.03162 | 0.02095 |
| 4911.58 | 4918.94 | 1953.42273 | 0.01054 | 0.00698 |
| 4918.94 | 4926.3 | 1953.32832 | 0.0155 | 0.01027 |
| 4926.3 | 4934.02 | 1953.28109 | 0.00744 | 0.00517 |
| 4934.02 | 4941.38 | 1953.13465 | 0.01798 | 0.01191 |
| 4941.38 | 4948.74 | 1953.04 | 0.0124 | 0.00822 |
| 4948.74 | 4956.1 | 1952.94527 | 0.02294 | 0.0152 |
| 4956.1 | 4963.46 | 1952.85046 | 0.01612 | 0.01068 |
| 4963.46 | 4970.82 | 1952.75557 | 0.01674 | 0.01109 |
| 4970.82 | 4978.18 | 1952.6606 | 0.01426 | 0.00945 |
| 4978.18 | 4985.54 | 1952.56555 | 0.0124 | 0.00822 |
| 4985.54 | 4992.9 | 1952.47042 | 0.0093 | 0.00616 |
| 4992.9 | 5000.26 | 1952.37522 | 0.01116 | 0.0074 |
| 5000.26 | 5007.62 | 1952.27993 | 0.01116 | 0.0074 |
| 5007.62 | 5014.98 | 1952.18456 | 0.01302 | 0.00863 |
| 5014.98 | 5022.7 | 1952.08677 | 0.00372 | 0.00259 |
| 5022.7 | 5030.06 | 1951.9889 | 0.00496 | 0.00329 |
| 5030.06 | 5037.42 | 1951.89329 | 0.02046 | 0.01356 |
| 5037.42 | 5044.78 | 1951.7976 | 0.01612 | 0.01068 |
| 5044.78 | 5052.14 | 1951.70182 | 6.20052E-4 | 4.1085E-4 |
| 5052.14 | 5059.5 | 1951.60597 | 0.01426 | 0.00945 |
| 5059.5 | 5067.13 | 1951.50834 | 0.01364 | 0.00937 |
| 5067.13 | 5074.44 | 1951.41089 | 0.0093 | 0.00612 |
| 5074.44 | 5081.75 | 1951.31545 | 0.01116 | 0.00735 |
| 5081.75 | 5089.06 | 1951.21992 | 0.01054 | 0.00694 |
| 5089.06 | 5096.37 | 1951.12432 | 0.01054 | 0.00694 |
| 5096.37 | 5103.68 | 1951.02864 | 0.01116 | 0.00735 |

**Table 4.** Sample intervals, estimated time of deposition, sulfate concentration, and calculated sulfate mass in core DH-98-4, Upper Fremont Glacier, Wyoming.

[cm, centimeters; SO_4_, sulfate; mg/L, milligrams per liter; kg/ha, kilograms per hectare; <, less than]

| Depth to top of sample interval, in cm | Depth to bottom of sample interval, in cm | Modeled age of ice core, in decimal years | SO_4_ concentration, in mg/L | Calculated SO_4_ mass, in kg/ha |
| --- | --- | --- | --- | --- |
| 623 | 630.36 | 1994.80969 | 0.07493 | 0.04965 |
| 630.36 | 637.72 | 1994.76192 | 0.09702 | 0.06429 |
| 637.72 | 645.08 | 1994.71408 | 0.08934 | 0.05919 |
| 645.08 | 652.44 | 1994.66615 | 0.05427 | 0.03596 |
| 652.44 | 659.8 | 1994.61815 | 0.03938 | 0.0261 |
| 659.8 | 667.16 | 1994.57007 | 0.02834 | 0.01878 |
| 667.16 | 674.52 | 1994.5219 | 0.07829 | 0.05188 |
| 674.52 | 681.88 | 1994.47366 | 0.05908 | 0.03915 |
| 681.88 | 689.24 | 1994.42533 | 0.07637 | 0.0506 |
| 689.24 | 696.6 | 1994.37693 | 0.06196 | 0.04105 |
| 696.6 | 703.96 | 1994.32844 | 0.05283 | 0.03501 |
| 703.96 | 711.32 | 1994.27988 | 0.07685 | 0.05092 |
| 711.32 | 718.68 | 1994.23123 | 0.03074 | 0.02037 |
| 718.68 | 726.04 | 1994.18251 | 0.02065 | 0.01368 |
| 726.04 | 733.4 | 1994.13371 | 0.03122 | 0.02069 |
| 733.4 | 740.76 | 1994.08482 | 0.04419 | 0.02928 |
| 740.76 | 748.12 | 1994.03586 | 0.04995 | 0.0331 |
| 748.12 | 755.48 | 1993.98681 | 0.02738 | 0.01814 |
| 755.48 | 762.84 | 1993.93769 | 0.02738 | 0.01814 |
| 762.84 | 770.2 | 1993.88848 | 0.03698 | 0.02451 |
| 770.2 | 777.56 | 1993.8392 | 0.04659 | 0.03087 |
| 777.56 | 784.92 | 1993.78983 | 0.0269 | 0.01782 |
| 784.92 | 792.28 | 1993.74038 | 0.02498 | 0.01655 |
| 792.28 | 799.64 | 1993.69086 | 0.02642 | 0.0175 |
| 799.64 | 807 | 1993.64125 | 0.0269 | 0.01782 |
| 807 | 816 | 1993.58603 | 0.04515 | 0.03658 |
| 816 | 830 | 1993.50822 | 0.08838 | 0.11139 |
| 830 | 841 | 1993.42342 | 0.04947 | 0.04899 |
| 841 | 852 | 1993.34861 | 0.05475 | 0.05422 |
| 852 | 863 | 1993.27362 | 0.06292 | 0.06231 |
| 863 | 874 | 1993.19846 | 0.05043 | 0.04994 |
| 874 | 885 | 1993.12311 | 0.04803 | 0.04756 |
| 885 | 896 | 1993.04758 | 0.02642 | 0.02616 |
| 896 | 907 | 1992.97188 | 0.02498 | 0.02473 |
| 907 | 918 | 1992.89599 | 0.02498 | 0.02473 |
| 918 | 929 | 1992.81993 | 0.01969 | 0.0195 |
| 929 | 940 | 1992.74369 | 0.02305 | 0.02283 |
| 940 | 951 | 1992.66727 | 0.03506 | 0.03472 |
| 951 | 962 | 1992.59067 | 0.0293 | 0.02901 |
| 962 | 973 | 1992.51389 | 0.02834 | 0.02806 |
| 973 | 981 | 1992.44744 | 0.03794 | 0.02733 |
| 981 | 992 | 1992.38085 | 0.03362 | 0.0333 |
| 992 | 1000 | 1992.31413 | 0.05571 | 0.04013 |
| 1000 | 1011 | 1992.24728 | 0.01873 | 0.01855 |
| 1011 | 1019.25 | 1992.17938 | 0.0245 | 0.01819 |
| 1019.25 | 1026.5 | 1992.12463 | 0.01969 | 0.01285 |
| 1026.5 | 1033.75 | 1992.07333 | < 0.01 | 0 |
| 1033.75 | 1041 | 1992.02196 | 0.01777 | 0.0116 |
| 1041 | 1048.25 | 1991.97051 | 0.01969 | 0.01285 |
| 1048.25 | 1055.5 | 1991.91898 | 0.01969 | 0.01285 |
| 1055.5 | 1062.75 | 1991.86738 | < 0.01 | 0 |
| 1062.75 | 1070 | 1991.81569 | < 0.01 | 0 |
| 1070 | 1077.2 | 1991.76415 | < 0.01 | 0 |
| 1077.2 | 1084.4 | 1991.71267 | < 0.01 | 0 |
| 1084.4 | 1091.6 | 1991.66111 | < 0.01 | 0 |
| 1091.6 | 1098.8 | 1991.60948 | < 0.01 | 0 |
| 1098.8 | 1106 | 1991.55776 | < 0.01 | 0 |
| 1106 | 1113.2 | 1991.50598 | < 0.01 | 0 |
| 1113.2 | 1120.4 | 1991.45411 | < 0.01 | 0 |
| 1120.4 | 1127.6 | 1991.40217 | < 0.01 | 0 |
| 1127.6 | 1134.8 | 1991.35016 | < 0.01 | 0 |
| 1134.8 | 1142 | 1991.29806 | < 0.01 | 0 |
| 1142 | 1149.2 | 1991.24589 | < 0.01 | 0 |
| 1149.2 | 1156.4 | 1991.19365 | < 0.01 | 0 |
| 1156.4 | 1163.6 | 1991.14132 | 0.04803 | 0.03113 |
| 1163.6 | 1170.8 | 1991.08892 | < 0.01 | 0 |
| 1170.8 | 1178 | 1991.03644 | 0.01633 | 0.01059 |
| 1178 | 1185.2 | 1990.98389 | < 0.01 | 0 |
| 1185.2 | 1192.4 | 1990.93126 | 0.02786 | 0.01806 |
| 1192.4 | 1199.6 | 1990.87855 | < 0.01 | 0 |
| 1199.6 | 1206.8 | 1990.82577 | 0.01681 | 0.0109 |
| 1206.8 | 1214 | 1990.77291 | < 0.01 | 0 |
| 1214 | 1224 | 1990.70967 | 0.07829 | 0.07048 |
| 1224 | 1234 | 1990.636 | 0.04419 | 0.03978 |
| 1234 | 1244 | 1990.56218 | 0.02738 | 0.02465 |
| 1244 | 1254 | 1990.48822 | < 0.01 | 0 |
| 1254 | 1264 | 1990.4141 | 0.02017 | 0.01816 |
| 1264 | 1274 | 1990.33984 | 0.01873 | 0.01686 |
| 1274 | 1284 | 1990.26543 | < 0.01 | 0 |
| 1284 | 1294 | 1990.19087 | 0.03266 | 0.0294 |
| 1294 | 1304 | 1990.11617 | 0.01873 | 0.01686 |
| 1304 | 1312 | 1990.04881 | 0.05139 | 0.03701 |
| 1312 | 1322 | 1989.98133 | 0.02305 | 0.02076 |
| 1322 | 1332 | 1989.90621 | 0.02305 | 0.02076 |
| 1332 | 1340 | 1989.83847 | < 0.01 | 0 |
| 1340 | 1348 | 1989.77817 | 0.02353 | 0.01695 |
| 1348 | 1356 | 1989.71776 | < 0.01 | 0 |
| 1356 | 1364 | 1989.65727 | < 0.01 | 0 |
| 1364 | 1372 | 1989.59667 | < 0.01 | 0 |
| 1372 | 1380 | 1989.53599 | 0.03362 | 0.02421 |
| 1380 | 1388 | 1989.47521 | 0.02065 | 0.01487 |
| 1388 | 1396 | 1989.41433 | 0.01873 | 0.01349 |
| 1396 | 1404 | 1989.35336 | 0.02017 | 0.01453 |
| 1404 | 1412 | 1989.2923 | 0.01873 | 0.01349 |
| 1412 | 1420 | 1989.23114 | 0.02065 | 0.01487 |
| 1420 | 1430 | 1989.16222 | 0.0269 | 0.02421 |
| 1430 | 1437.8 | 1989.09395 | 0.02065 | 0.0145 |
| 1437.8 | 1445.6 | 1989.03402 | 0.03074 | 0.02159 |
| 1445.6 | 1453.4 | 1988.974 | 0.01777 | 0.01248 |
| 1453.4 | 1461.2 | 1988.9139 | < 0.01 | 0 |
| 1461.2 | 1469 | 1988.8537 | 0.01873 | 0.01315 |
| 1469 | 1476.8 | 1988.79341 | < 0.01 | 0 |
| 1476.8 | 1484.6 | 1988.73303 | < 0.01 | 0 |
| 1484.6 | 1492.4 | 1988.67256 | 0.01777 | 0.01248 |
| 1492.4 | 1500.2 | 1988.61201 | 0.04755 | 0.03339 |
| 1500.2 | 1508 | 1988.55136 | 0.05475 | 0.03845 |
| 1508 | 1516.4 | 1988.48829 | 0.04947 | 0.03741 |
| 1516.4 | 1524.2 | 1988.42511 | 0.08597 | 0.06037 |
| 1524.2 | 1532 | 1988.36419 | 0.0927 | 0.06509 |
| 1532 | 1539.8 | 1988.30318 | 0.09846 | 0.06914 |
| 1539.8 | 1547.6 | 1988.24207 | 0.08405 | 0.05902 |
| 1547.6 | 1555.4 | 1988.18088 | 0.08261 | 0.05801 |
| 1555.4 | 1563.2 | 1988.11959 | 0.04995 | 0.03508 |
| 1563.2 | 1571 | 1988.05822 | 0.03842 | 0.02698 |
| 1571 | 1578.8 | 1987.99676 | 0.07637 | 0.05363 |
| 1578.8 | 1586.6 | 1987.9352 | 0.08261 | 0.05801 |
| 1586.6 | 1594.4 | 1987.87356 | 0.06388 | 0.04486 |
| 1594.4 | 1602.2 | 1987.81183 | 0.08069 | 0.05666 |
| 1602.2 | 1610 | 1987.75 | 0.05764 | 0.04047 |
| 1610 | 1620 | 1987.67935 | < 0.01 | 0 |
| 1620 | 1628.6 | 1987.6054 | < 0.01 | 0 |
| 1628.6 | 1636.8 | 1987.53849 | < 0.01 | 0 |
| 1636.8 | 1644.4 | 1987.47548 | < 0.01 | 0 |
| 1644.4 | 1652 | 1987.41476 | < 0.01 | 0 |
| 1652 | 1659.6 | 1987.35397 | < 0.01 | 0 |
| 1659.6 | 1667.2 | 1987.29308 | < 0.01 | 0 |
| 1667.2 | 1674.8 | 1987.23212 | < 0.01 | 0 |
| 1674.8 | 1683 | 1987.16865 | < 0.01 | 0 |
| 1683 | 1690.6 | 1987.10509 | < 0.01 | 0 |
| 1690.6 | 1698.2 | 1987.04386 | 0.0389 | 0.02662 |
| 1698.2 | 1705.8 | 1986.98255 | 0.06676 | 0.04568 |
| 1705.8 | 1713.4 | 1986.92114 | 0.03938 | 0.02695 |
| 1713.4 | 1721 | 1986.85966 | 0.04323 | 0.02958 |
| 1721 | 1728.6 | 1986.79808 | 0.02882 | 0.01972 |
| 1728.6 | 1736.2 | 1986.73643 | 0.06724 | 0.04601 |
| 1736.2 | 1743.8 | 1986.67468 | 0.04659 | 0.03188 |
| 1743.8 | 1751.4 | 1986.61286 | 0.11287 | 0.07723 |
| 1751.4 | 1759 | 1986.55094 | 0.08693 | 0.05948 |
| 1759 | 1770.2 | 1986.47425 | 0.04275 | 0.0431 |
| 1770.2 | 1780.8 | 1986.38515 | 0.061 | 0.05821 |
| 1780.8 | 1791.4 | 1986.29833 | 0.02065 | 0.01971 |
| 1791.4 | 1801.4 | 1986.21382 | 0.09414 | 0.08475 |
| 1801.4 | 1811.4 | 1986.13161 | 0.19644 | 0.17685 |
| 1811.4 | 1821.4 | 1986.04926 | 0.7387 | 0.66504 |
| 1821.4 | 1831.2 | 1985.96758 | 0.08309 | 0.07331 |
| 1831.2 | 1838.6 | 1985.89652 | 0.09942 | 0.06624 |
| 1838.6 | 1846 | 1985.83528 | 0.25696 | 0.17119 |
| 1846 | 1853.8 | 1985.7723 | 0.04563 | 0.03204 |
| 1853.8 | 1861.2 | 1985.70924 | 0.06004 | 0.04 |
| 1861.2 | 1868.6 | 1985.64775 | 0.06052 | 0.04032 |
| 1868.6 | 1876 | 1985.58619 | 0.02882 | 0.0192 |
| 1876 | 1883.4 | 1985.52454 | 0.02642 | 0.0176 |
| 1883.4 | 1890.8 | 1985.46281 | 0.17579 | 0.11711 |
| 1890.8 | 1898.8 | 1985.39849 | 0.03122 | 0.02249 |
| 1898.8 | 1906.2 | 1985.33409 | 0.03362 | 0.0224 |
| 1906.2 | 1913.6 | 1985.27211 | 0.02882 | 0.0192 |
| 1913.6 | 1921 | 1985.21005 | 0.0293 | 0.01952 |
| 1921 | 1928.4 | 1985.14791 | 0.03746 | 0.02496 |
| 1928.4 | 1935.8 | 1985.08569 | 0.03314 | 0.02208 |
| 1935.8 | 1943.6 | 1985.02171 | 0.03602 | 0.0253 |
| 1943.6 | 1951 | 1984.95764 | 0.02257 | 0.01504 |
| 1951 | 1958.4 | 1984.89517 | 0.0317 | 0.02112 |
| 1958.4 | 1965.8 | 1984.83262 | 0.04467 | 0.02976 |
| 1965.8 | 1973.2 | 1984.76999 | 0.04947 | 0.03296 |
| 1973.2 | 1980.6 | 1984.70728 | 0.03266 | 0.02176 |
| 1980.6 | 1988.45 | 1984.64253 | 0.0634 | 0.04481 |
| 1988.45 | 1995.9 | 1984.57753 | 0.06772 | 0.04542 |
| 1995.9 | 2003.35 | 1984.51415 | 0.03794 | 0.02545 |
| 2003.35 | 2010.8 | 1984.45068 | 0.02786 | 0.01868 |
| 2010.8 | 2018.25 | 1984.38714 | 0.01969 | 0.01321 |
| 2018.25 | 2025.7 | 1984.32351 | 0.07925 | 0.05315 |
| 2025.7 | 2034.6 | 1984.25791 | 0.0245 | 0.01963 |
| 2034.6 | 2042.05 | 1984.19214 | 0.03074 | 0.02062 |
| 2042.05 | 2049.5 | 1984.12826 | 0.02209 | 0.01482 |
| 2049.5 | 2056.95 | 1984.0643 | 0.0269 | 0.01804 |
| 2056.95 | 2064.4 | 1984.00025 | 0.03122 | 0.02094 |
| 2064.4 | 2071.85 | 1983.93612 | 0.01921 | 0.01289 |
| 2071.85 | 2079.3 | 1983.87192 | 0.03026 | 0.0203 |
| 2079.3 | 2086.75 | 1983.80762 | 0.04179 | 0.02803 |
| 2086.75 | 2094.2 | 1983.74325 | 0.21902 | 0.1469 |
| 2094.2 | 2101.65 | 1983.67879 | 0.02161 | 0.0145 |
| 2101.65 | 2109.1 | 1983.61426 | 0.01873 | 0.01256 |
| 2109.1 | 2116.55 | 1983.54964 | 0.02594 | 0.0174 |
| 2116.55 | 2124.45 | 1983.48302 | 0.0341 | 0.02425 |
| 2124.45 | 2131.9 | 1983.41624 | 0.02738 | 0.01836 |
| 2131.9 | 2139.35 | 1983.35137 | 0.08309 | 0.05573 |
| 2139.35 | 2146.8 | 1983.28641 | 0.02882 | 0.01933 |
| 2146.8 | 2154.7 | 1983.21946 | 0.0903 | 0.06422 |
| 2154.7 | 2162.15 | 1983.15233 | 0.01873 | 0.01256 |
| 2162.15 | 2169.6 | 1983.08712 | 0.07301 | 0.04897 |
| 2169.6 | 2177.05 | 1983.02184 | 0.0365 | 0.02448 |
| 2177.05 | 2184.5 | 1982.95647 | 0.01633 | 0.01095 |
| 2184.5 | 2194.75 | 1982.87871 | 0.08501 | 0.07845 |
| 2194.75 | 2205.67 | 1982.7856 | 0.03122 | 0.03069 |
| 2205.67 | 2215.81 | 1982.69276 | 0.05571 | 0.05086 |
| 2215.81 | 2223.28 | 1982.61497 | 0.06628 | 0.04458 |
| 2223.28 | 2230.75 | 1982.54891 | 0.03314 | 0.02229 |
| 2230.75 | 2238.22 | 1982.48278 | 0.07829 | 0.05265 |
| 2238.22 | 2245.69 | 1982.41656 | 0.33045 | 0.22223 |
| 2245.69 | 2253.16 | 1982.35026 | 0.06772 | 0.04554 |
| 2253.16 | 2260.63 | 1982.28387 | 0.03266 | 0.02196 |
| 2260.63 | 2268.1 | 1982.21741 | 0.03314 | 0.02229 |
| 2268.1 | 2275.57 | 1982.15086 | 0.04131 | 0.02778 |
| 2275.57 | 2283.04 | 1982.08423 | 0.09414 | 0.06331 |
| 2283.04 | 2290.98 | 1982.01546 | 0.07445 | 0.05322 |
| 2290.98 | 2298.45 | 1981.94651 | 0.04563 | 0.03069 |
| 2298.45 | 2305.92 | 1981.87963 | 0.0245 | 0.01647 |
| 2305.92 | 2313.39 | 1981.81266 | 0.03122 | 0.021 |
| 2313.39 | 2320.86 | 1981.7457 | 0.10903 | 0.07332 |
| 2320.86 | 2328.33 | 1981.67857 | 0.01537 | 0.01034 |
| 2328.33 | 2335.8 | 1981.61136 | 0.01969 | 0.01324 |
| 2335.8 | 2343.27 | 1981.54406 | 0.01489 | 0.01001 |
| 2343.27 | 2350.74 | 1981.47669 | 0.08069 | 0.05427 |
| 2350.74 | 2358.21 | 1981.40922 | 0.01969 | 0.01324 |
| 2358.21 | 2365.68 | 1981.34168 | 0.02882 | 0.01938 |
| 2365.68 | 2373.15 | 1981.27406 | 0.03026 | 0.02035 |
| 2373.15 | 2381.09 | 1981.20417 | 0.01969 | 0.01408 |
| 2381.09 | 2388.56 | 1981.13429 | 0.01633 | 0.01098 |
| 2388.56 | 2396.03 | 1981.06641 | 0.04083 | 0.02746 |
| 2396.03 | 2403.6 | 1980.99799 | 0.0389 | 0.02651 |
| 2403.6 | 2410.63 | 1980.9315 | 0.01297 | 0.00821 |
| 2410.63 | 2417.66 | 1980.86739 | 0.03026 | 0.01915 |
| 2417.66 | 2424.69 | 1980.80321 | 0.01105 | 0.00699 |
| 2424.69 | 2431.72 | 1980.73895 | 0.00865 | 0.00547 |
| 2431.72 | 2438.75 | 1980.67462 | 0.01249 | 0.0079 |
| 2438.75 | 2445.78 | 1980.61022 | 0.02305 | 0.01459 |
| 2445.78 | 2452.81 | 1980.54575 | < 0.01 | 0 |
| 2452.81 | 2459.84 | 1980.4812 | 0.01585 | 0.01003 |
| 2459.84 | 2466.9 | 1980.41639 | 0.02113 | 0.01343 |
| 2466.9 | 2473.93 | 1980.35161 | 0.01105 | 0.00699 |
| 2473.93 | 2480.96 | 1980.28684 | 0.01441 | 0.00912 |
| 2480.96 | 2487.99 | 1980.222 | 0.00961 | 0.00608 |
| 2487.99 | 2495.02 | 1980.15709 | 0.01249 | 0.0079 |
| 2495.02 | 2502.05 | 1980.09201 | 0.03026 | 0.01915 |
| 2502.05 | 2508.58 | 1980.02926 | 0.05283 | 0.03106 |
| 2508.58 | 2516.11 | 1979.96413 | 0.01441 | 0.00977 |
| 2516.11 | 2523.14 | 1979.89661 | 0.02257 | 0.01429 |
| 2523.14 | 2530.17 | 1979.83133 | 0.01681 | 0.01064 |
| 2530.17 | 2537.2 | 1979.76598 | 0.01345 | 0.00851 |
| 2537.2 | 2544.23 | 1979.70055 | 0.01153 | 0.0073 |
| 2544.23 | 2551.26 | 1979.63506 | 0.0389 | 0.02462 |
| 2551.26 | 2558.32 | 1979.56939 | 0.03026 | 0.01923 |
| 2558.32 | 2565.35 | 1979.50356 | 0.01345 | 0.00851 |
| 2565.35 | 2572.38 | 1979.43784 | 0.03554 | 0.02249 |
| 2572.38 | 2579.41 | 1979.37205 | 0.0269 | 0.01702 |
| 2579.41 | 2586.44 | 1979.30619 | 0.02161 | 0.01368 |
| 2586.44 | 2593.47 | 1979.24025 | 0.01489 | 0.00942 |
| 2593.47 | 2600.55 | 1979.17406 | 0.02209 | 0.01408 |
| 2600.55 | 2607.6 | 1979.1076 | 0.05139 | 0.03262 |
| 2607.6 | 2614.65 | 1979.04125 | 0.0586 | 0.03719 |
| 2614.65 | 2621.7 | 1978.97484 | 0.02305 | 0.01463 |
| 2621.7 | 2628.75 | 1978.90834 | 0.02978 | 0.0189 |
| 2628.75 | 2635.8 | 1978.84178 | 0.01681 | 0.01067 |
| 2635.8 | 2642.85 | 1978.77514 | 0.0269 | 0.01707 |
| 2642.85 | 2649.9 | 1978.70843 | 0.03938 | 0.025 |
| 2649.9 | 2657.12 | 1978.64089 | 0.01201 | 0.0078 |
| 2657.12 | 2664.14 | 1978.57336 | 0.01873 | 0.01184 |
| 2664.14 | 2671.16 | 1978.50671 | 0.0269 | 0.017 |
| 2671.16 | 2678.18 | 1978.43999 | 0.01777 | 0.01123 |
| 2678.18 | 2685.2 | 1978.3732 | 0.01681 | 0.01062 |
| 2685.2 | 2692.22 | 1978.30633 | 0.04563 | 0.02884 |
| 2692.22 | 2699.24 | 1978.23939 | 0.05283 | 0.03339 |
| 2699.24 | 2706.26 | 1978.17238 | 0.01825 | 0.01153 |
| 2706.26 | 2713.28 | 1978.10529 | 0.04611 | 0.02914 |
| 2713.28 | 2720.3 | 1978.03813 | 0.01633 | 0.01032 |
| 2720.3 | 2727.34 | 1977.97081 | 0.01777 | 0.01126 |
| 2727.34 | 2734.36 | 1977.90341 | 0.03266 | 0.02064 |
| 2734.36 | 2741.38 | 1977.83603 | 0.0317 | 0.02003 |
| 2741.38 | 2748.4 | 1977.76858 | 0.04899 | 0.03096 |
| 2748.4 | 2755.42 | 1977.70106 | 0.01297 | 0.0082 |
| 2755.42 | 2762.44 | 1977.63346 | 0.02065 | 0.01305 |
| 2762.44 | 2769.46 | 1977.56579 | 0.04467 | 0.02823 |
| 2769.46 | 2776.48 | 1977.49805 | 0.04659 | 0.02944 |
| 2776.48 | 2783.57 | 1977.42985 | 0.01153 | 0.00736 |
| 2783.57 | 2790.7 | 1977.36109 | 0.24351 | 0.15631 |
| 2790.7 | 2797.83 | 1977.29206 | 0.03122 | 0.02004 |
| 2797.83 | 2804.96 | 1977.22296 | 0.01441 | 0.00925 |
| 2804.96 | 2812.09 | 1977.15378 | 0.03266 | 0.02096 |
| 2812.09 | 2819.35 | 1977.08395 | 0.0341 | 0.02229 |
| 2819.35 | 2826.48 | 1977.01394 | 0.07349 | 0.04717 |
| 2826.48 | 2833.61 | 1976.94454 | 0.02161 | 0.01387 |
| 2833.61 | 2840.74 | 1976.87506 | 0.01921 | 0.01233 |
| 2840.74 | 2847.87 | 1976.8055 | 0.02209 | 0.01418 |
| 2847.87 | 2854.86 | 1976.73656 | 0.02113 | 0.0133 |
| 2854.86 | 2861.82 | 1976.66842 | 0.01681 | 0.01053 |
| 2861.82 | 2868.78 | 1976.60031 | 0.00624 | 0.00391 |
| 2868.78 | 2875.74 | 1976.53212 | 0.01201 | 0.00752 |
| 2875.74 | 2882.7 | 1976.46387 | 0.01393 | 0.00873 |
| 2882.7 | 2889.66 | 1976.39554 | 0.01249 | 0.00782 |
| 2889.66 | 2896.62 | 1976.32714 | 0.03458 | 0.02167 |
| 2896.62 | 2903.58 | 1976.25867 | 0.03602 | 0.02257 |
| 2903.58 | 2910.54 | 1976.19013 | 0.02305 | 0.01445 |
| 2910.54 | 2917.5 | 1976.12151 | 0.01969 | 0.01234 |
| 2917.5 | 2924.46 | 1976.05283 | 0.01681 | 0.01053 |
| 2924.46 | 2931.82 | 1975.98209 | 0.01873 | 0.01241 |
| 2931.82 | 2938.78 | 1975.91128 | 0.02209 | 0.01384 |
| 2938.78 | 2945.74 | 1975.84238 | 0.04947 | 0.031 |
| 2945.74 | 2952.7 | 1975.7734 | 0.01729 | 0.01083 |
| 2952.7 | 2959.66 | 1975.70436 | 0.01681 | 0.01053 |
| 2959.66 | 2966.62 | 1975.63524 | 0.01297 | 0.00813 |
| 2966.62 | 2973.58 | 1975.56605 | 0.05379 | 0.03371 |
| 2973.58 | 2980.54 | 1975.49678 | 0.02834 | 0.01776 |
| 2980.54 | 2988.1 | 1975.42446 | 0.01585 | 0.01079 |
| 2988.1 | 2995.55 | 1975.34956 | 0.05764 | 0.03866 |
| 2995.55 | 3003 | 1975.27518 | 0.01777 | 0.01192 |
| 3003 | 3010.45 | 1975.20072 | 0.01345 | 0.00902 |
| 3010.45 | 3017.9 | 1975.12618 | 0.02546 | 0.01707 |
| 3017.9 | 3025.35 | 1975.05155 | 0.01777 | 0.01192 |
| 3025.35 | 3032.8 | 1974.97684 | 0.03314 | 0.02223 |
| 3032.8 | 3040.25 | 1974.90205 | 0.01249 | 0.00838 |
| 3040.25 | 3047.7 | 1974.82718 | 0.02065 | 0.01385 |
| 3047.7 | 3055.15 | 1974.75222 | 0.01201 | 0.00805 |
| 3055.15 | 3062.6 | 1974.67719 | 0.02161 | 0.0145 |
| 3062.6 | 3070.05 | 1974.60207 | 0.01345 | 0.00902 |
| 3070.05 | 3077.95 | 1974.52465 | 0.03506 | 0.02494 |
| 3077.95 | 3085.85 | 1974.44481 | 0.01297 | 0.00922 |
| 3085.85 | 3093.3 | 1974.36711 | 0.0293 | 0.01965 |
| 3093.3 | 3100.75 | 1974.29165 | 0.17627 | 0.11823 |
| 3100.75 | 3108.2 | 1974.21611 | 0.02017 | 0.01353 |
| 3108.2 | 3116.1 | 1974.13826 | 0.01633 | 0.01161 |
| 3116.1 | 3123.55 | 1974.06022 | 0.00913 | 0.00612 |
| 3123.55 | 3131.33 | 1973.9828 | 0.02738 | 0.01918 |
| 3131.33 | 3138.62 | 1973.90601 | 0.01489 | 0.00977 |
| 3138.62 | 3145.91 | 1973.83168 | 0.02402 | 0.01576 |
| 3145.91 | 3153.2 | 1973.75728 | 0.01825 | 0.01198 |
| 3153.2 | 3160.49 | 1973.6828 | 0.02353 | 0.01545 |
| 3160.49 | 3167.78 | 1973.60824 | 0.02017 | 0.01324 |
| 3167.78 | 3175.07 | 1973.5336 | 0.01777 | 0.01166 |
| 3175.07 | 3182.36 | 1973.45888 | 0.0245 | 0.01608 |
| 3182.36 | 3189.65 | 1973.38409 | 0.02402 | 0.01576 |
| 3189.65 | 3196.94 | 1973.30921 | 0.02546 | 0.01671 |
| 3196.94 | 3204.23 | 1973.23426 | 0.01921 | 0.01261 |
| 3204.23 | 3211.52 | 1973.15923 | 0.02065 | 0.01355 |
| 3211.52 | 3218.81 | 1973.08412 | 0.01729 | 0.01135 |
| 3218.81 | 3226.1 | 1973.00893 | 0.01537 | 0.01009 |
| 3226.1 | 3233.39 | 1972.93366 | 0.01441 | 0.00946 |
| 3233.39 | 3240.68 | 1972.85832 | 0.05091 | 0.03341 |
| 3240.68 | 3247.97 | 1972.78289 | 0.01633 | 0.01072 |
| 3247.97 | 3255.55 | 1972.70594 | 0.01153 | 0.00787 |
| 3255.55 | 3262.84 | 1972.6288 | 0.01585 | 0.0104 |
| 3262.84 | 3270.13 | 1972.55314 | 0.01441 | 0.00946 |
| 3270.13 | 3277.42 | 1972.47739 | 0.01585 | 0.0104 |
| 3277.42 | 3284.71 | 1972.40157 | 0.01681 | 0.01103 |
| 3284.71 | 3292.64 | 1972.32234 | 0.01969 | 0.01406 |
| 3292.64 | 3300.26 | 1972.24136 | 0.01345 | 0.00923 |
| 3300.26 | 3307.88 | 1972.16185 | 0.02642 | 0.01812 |
| 3307.88 | 3315.5 | 1972.08225 | 0.01729 | 0.01186 |
| 3315.5 | 3323.12 | 1972.00257 | 0.02017 | 0.01384 |
| 3323.12 | 3330.74 | 1971.9228 | 0.02065 | 0.01417 |
| 3330.74 | 3338.36 | 1971.84295 | 0.01441 | 0.00988 |
| 3338.36 | 3345.98 | 1971.76301 | 0.01441 | 0.00988 |
| 3345.98 | 3353.6 | 1971.68299 | 0.01393 | 0.00956 |
| 3353.6 | 3361.22 | 1971.60288 | 0.01393 | 0.00956 |
| 3361.22 | 3368.84 | 1971.52268 | 0.01585 | 0.01087 |
| 3368.84 | 3376.46 | 1971.4424 | 0.01537 | 0.01054 |
| 3376.46 | 3384.08 | 1971.36203 | 0.01297 | 0.0089 |
| 3384.08 | 3391.7 | 1971.28158 | 0.02978 | 0.02043 |
| 3391.7 | 3399.32 | 1971.20104 | 0.03746 | 0.0257 |
| 3399.32 | 3406.94 | 1971.12041 | 0.34726 | 0.23822 |
| 3406.94 | 3414.56 | 1971.0397 | 0.01585 | 0.01087 |
| 3414.56 | 3422.18 | 1970.95891 | 0.01009 | 0.00692 |
| 3422.18 | 3429.8 | 1970.87802 | 0.02017 | 0.01384 |
| 3429.8 | 3438.04 | 1970.79376 | 0.02257 | 0.01675 |
| 3438.04 | 3445.66 | 1970.7094 | 0.04323 | 0.02965 |
| 3445.66 | 3453.28 | 1970.62826 | 0.01009 | 0.00692 |
| 3453.28 | 3460.9 | 1970.54702 | 0.01489 | 0.01021 |
| 3460.9 | 3469.14 | 1970.46239 | 0.01873 | 0.0139 |
| 3469.14 | 3476.76 | 1970.37767 | 0.01873 | 0.01285 |
| 3476.76 | 3484.38 | 1970.29618 | 0.02402 | 0.01647 |
| 3484.38 | 3492.56 | 1970.21159 | 0.01825 | 0.01344 |
| 3492.56 | 3499.92 | 1970.12831 | 0.01393 | 0.00923 |
| 3499.92 | 3507.28 | 1970.04934 | 0.01393 | 0.00923 |
| 3507.28 | 3514.64 | 1969.9703 | 0.01153 | 0.00764 |
| 3514.64 | 3522.36 | 1969.88923 | 0.03938 | 0.02737 |
| 3522.36 | 3529.72 | 1969.80808 | 0.01537 | 0.01018 |
| 3529.72 | 3537.08 | 1969.72879 | 0.02065 | 0.01368 |
| 3537.08 | 3544.44 | 1969.64942 | 0.04899 | 0.03246 |
| 3544.44 | 3551.8 | 1969.56996 | 0.01393 | 0.00923 |
| 3551.8 | 3559.16 | 1969.49043 | 0.01681 | 0.01114 |
| 3559.16 | 3566.52 | 1969.41082 | 0.03122 | 0.02069 |
| 3566.52 | 3573.88 | 1969.33113 | 0.01681 | 0.01114 |
| 3573.88 | 3581.24 | 1969.25135 | 0.01969 | 0.01305 |
| 3581.24 | 3588.6 | 1969.1715 | 0.01969 | 0.01305 |
| 3588.6 | 3595.96 | 1969.09157 | 0.02113 | 0.014 |
| 3595.96 | 3603.32 | 1969.01155 | 0.04083 | 0.02705 |
| 3603.32 | 3611.04 | 1968.9295 | 0.03026 | 0.02103 |
| 3611.04 | 3618.4 | 1968.84736 | 0.02546 | 0.01687 |
| 3618.4 | 3625.76 | 1968.76711 | 0.01345 | 0.00891 |
| 3625.76 | 3633.12 | 1968.68677 | 0.01825 | 0.01209 |
| 3633.12 | 3640.48 | 1968.60635 | 0.01969 | 0.01305 |
| 3640.48 | 3647.84 | 1968.52585 | 0.01201 | 0.00796 |
| 3647.84 | 3655.2 | 1968.44528 | 0.01201 | 0.00796 |
| 3655.2 | 3662.56 | 1968.36462 | 0.02353 | 0.01559 |
| 3662.56 | 3670.28 | 1968.28191 | 0.01681 | 0.01168 |
| 3670.28 | 3677.64 | 1968.19911 | 0.01969 | 0.01305 |
| 3677.64 | 3686.72 | 1968.10875 | 0.01969 | 0.0161 |
| 3686.72 | 3694.36 | 1968.01675 | 0.04419 | 0.03039 |
| 3694.36 | 3703.64 | 1967.92354 | 0.01777 | 0.01485 |
| 3703.64 | 3711.28 | 1967.83023 | 0.02113 | 0.01454 |
| 3711.28 | 3718.92 | 1967.74587 | 0.01441 | 0.00991 |
| 3718.92 | 3726.56 | 1967.66142 | 0.01969 | 0.01354 |
| 3726.56 | 3734.2 | 1967.57689 | 0.01153 | 0.00793 |
| 3734.2 | 3741.84 | 1967.49227 | 0.02978 | 0.02048 |
| 3741.84 | 3749.48 | 1967.40757 | 0.01393 | 0.00958 |
| 3749.48 | 3757.12 | 1967.32278 | 0.01633 | 0.01123 |
| 3757.12 | 3764.76 | 1967.2379 | 0.01921 | 0.01321 |
| 3764.76 | 3772.4 | 1967.15294 | 0.01537 | 0.01057 |
| 3772.4 | 3780.04 | 1967.06789 | 0.01009 | 0.00694 |
| 3780.04 | 3787.68 | 1966.98275 | 0.01585 | 0.0109 |
| 3787.68 | 3795.32 | 1966.89753 | 0.01105 | 0.0076 |
| 3795.32 | 3802.96 | 1966.81222 | 0.01585 | 0.0109 |
| 3802.96 | 3810.6 | 1966.72683 | 0.01537 | 0.01057 |
| 3810.6 | 3818.24 | 1966.64135 | 0.01633 | 0.01123 |
| 3818.24 | 3825.88 | 1966.55578 | 0.01345 | 0.00925 |
| 3825.88 | 3833.52 | 1966.47013 | 0.01825 | 0.01255 |
| 3833.52 | 3841.16 | 1966.38439 | 0.00913 | 0.00628 |
| 3841.16 | 3848.8 | 1966.29856 | 0.00576 | 0.00396 |
| 3848.8 | 3856.44 | 1966.21265 | 0.01345 | 0.00925 |
| 3856.44 | 3864.08 | 1966.12665 | 0.01009 | 0.00694 |
| 3864.08 | 3871.72 | 1966.04057 | 0.01489 | 0.01024 |
| 3871.72 | 3883 | 1965.93385 | 0.02065 | 0.02097 |
| 3883 | 3890.32 | 1965.82882 | 0.01057 | 0.00696 |
| 3890.32 | 3897.63 | 1965.74617 | 0.01297 | 0.00853 |
| 3897.63 | 3904.94 | 1965.66344 | 0.01441 | 0.00948 |
| 3904.94 | 3912.25 | 1965.58063 | 0.01249 | 0.00822 |
| 3912.25 | 3919.56 | 1965.49775 | 0.00865 | 0.00569 |
| 3919.56 | 3926.87 | 1965.41478 | 0.00768 | 0.00506 |
| 3926.87 | 3934.18 | 1965.33173 | 0.01057 | 0.00695 |
| 3934.18 | 3941.49 | 1965.24861 | 0.01393 | 0.00917 |
| 3941.49 | 3948.8 | 1965.16541 | 0.02113 | 0.01391 |
| 3948.8 | 3956.11 | 1965.08213 | 0.00961 | 0.00632 |
| 3956.11 | 3963.73 | 1964.99694 | 0.00817 | 0.0056 |
| 3963.73 | 3971.04 | 1964.91179 | 0.00672 | 0.00443 |
| 3971.04 | 3978.35 | 1964.82826 | 0.00865 | 0.00569 |
| 3978.35 | 3985.66 | 1964.74466 | 0.01009 | 0.00664 |
| 3985.66 | 3992.97 | 1964.66098 | 0.01057 | 0.00695 |
| 3992.97 | 4000.28 | 1964.57722 | 0.01297 | 0.00853 |
| 4000.28 | 4007.59 | 1964.49339 | 0.00961 | 0.00632 |
| 4007.59 | 4014.9 | 1964.40947 | 0.00913 | 0.00601 |
| 4014.9 | 4022.21 | 1964.32547 | 0.01345 | 0.00885 |
| 4022.21 | 4029.52 | 1964.2414 | 0.01057 | 0.00695 |
| 4029.52 | 4036.83 | 1964.15725 | 0.01585 | 0.01043 |
| 4036.83 | 4044.14 | 1964.07301 | 0.00961 | 0.00632 |
| 4044.14 | 4051.76 | 1963.98685 | 0.00865 | 0.00593 |
| 4051.76 | 4059.07 | 1963.90073 | 0.01441 | 0.00948 |
| 4059.07 | 4066.38 | 1963.81626 | 0.00961 | 0.00632 |
| 4066.38 | 4073.69 | 1963.7317 | 0.01105 | 0.00727 |
| 4073.69 | 4081.3 | 1963.64534 | 0.00624 | 0.00428 |
| 4081.3 | 4088.7 | 1963.55831 | 0.01057 | 0.00704 |
| 4088.7 | 4096.1 | 1963.47247 | 0.00336 | 0.00224 |
| 4096.1 | 4103.5 | 1963.38655 | 0.00961 | 0.0064 |
| 4103.5 | 4110.9 | 1963.30055 | 0.01057 | 0.00704 |
| 4110.9 | 4118.3 | 1963.21447 | 0.00528 | 0.00352 |
| 4118.3 | 4125.7 | 1963.12831 | 0.01201 | 0.008 |
| 4125.7 | 4133.1 | 1963.04207 | 0.00913 | 0.00608 |
| 4133.1 | 4140.9 | 1962.95341 | 0.01777 | 0.01248 |
| 4140.9 | 4148.3 | 1962.86467 | 0.00913 | 0.00608 |
| 4148.3 | 4155.7 | 1962.77818 | 0.01105 | 0.00736 |
| 4155.7 | 4163.1 | 1962.69161 | 0.01009 | 0.00672 |
| 4163.1 | 4170.5 | 1962.60496 | 0.00432 | 0.00288 |
| 4170.5 | 4177.9 | 1962.51823 | 0.00913 | 0.00608 |
| 4177.9 | 4185.3 | 1962.43141 | 0.00528 | 0.00352 |
| 4185.3 | 4192.7 | 1962.34452 | 0.00672 | 0.00448 |
| 4192.7 | 4200.1 | 1962.25755 | 0.00865 | 0.00576 |
| 4200.1 | 4207.5 | 1962.17049 | 0.00768 | 0.00512 |
| 4207.5 | 4214.9 | 1962.08335 | 0.00624 | 0.00416 |
| 4214.9 | 4222.3 | 1961.99614 | 0.01201 | 0.008 |
| 4222.3 | 4230.1 | 1961.90648 | 0.01201 | 0.00843 |
| 4230.1 | 4237.5 | 1961.81673 | 0.01201 | 0.008 |
| 4237.5 | 4244.9 | 1961.72927 | 0.05427 | 0.03616 |
| 4244.9 | 4252.3 | 1961.64172 | 0.03458 | 0.02304 |
| 4252.3 | 4259.7 | 1961.5541 | 0.03026 | 0.02016 |
| 4259.7 | 4267.1 | 1961.46639 | 0.01873 | 0.01248 |
| 4267.1 | 4274.92 | 1961.37611 | 0.01825 | 0.01285 |
| 4274.92 | 4282.28 | 1961.28598 | 0.03218 | 0.02132 |
| 4282.28 | 4289.64 | 1961.1985 | 0.01153 | 0.00764 |
| 4289.64 | 4297 | 1961.11094 | 0.01201 | 0.00796 |
| 4297 | 4304.36 | 1961.0233 | 0.00961 | 0.00637 |
| 4304.36 | 4311.72 | 1960.93558 | 0.00672 | 0.00446 |
| 4311.72 | 4319.44 | 1960.84564 | 0.01201 | 0.00835 |
| 4319.44 | 4326.8 | 1960.7556 | 0.01201 | 0.00796 |
| 4326.8 | 4334.16 | 1960.66764 | 0.01537 | 0.01018 |
| 4334.16 | 4341.52 | 1960.5796 | 0.01921 | 0.01273 |
| 4341.52 | 4348.88 | 1960.49147 | 0.04611 | 0.03055 |
| 4348.88 | 4356.24 | 1960.40327 | 0.01201 | 0.00796 |
| 4356.24 | 4363.6 | 1960.31499 | 0.01249 | 0.00827 |
| 4363.6 | 4370.96 | 1960.22662 | 0.01729 | 0.01146 |
| 4370.96 | 4378.32 | 1960.13818 | 0.01153 | 0.00764 |
| 4378.32 | 4385.68 | 1960.04966 | 0.01105 | 0.00732 |
| 4385.68 | 4393.04 | 1959.96105 | 0.00865 | 0.00573 |
| 4393.04 | 4400.4 | 1959.87237 | 0.01393 | 0.00923 |
| 4400.4 | 4408.12 | 1959.78143 | 0.01681 | 0.01168 |
| 4408.12 | 4415.48 | 1959.69041 | 0.01249 | 0.00827 |
| 4415.48 | 4422.84 | 1959.60148 | 0.01585 | 0.0105 |
| 4422.84 | 4430.2 | 1959.51248 | 0.02257 | 0.01496 |
| 4430.2 | 4437.56 | 1959.42339 | 0.02113 | 0.014 |
| 4437.56 | 4444.92 | 1959.33422 | 0.01153 | 0.00764 |
| 4444.92 | 4452.28 | 1959.24497 | 0.01009 | 0.00668 |
| 4452.28 | 4459.64 | 1959.15564 | 0.01681 | 0.01114 |
| 4459.64 | 4467 | 1959.06624 | 0.03986 | 0.02641 |
| 4467 | 4477.16 | 1958.95972 | 0.01873 | 0.01713 |
| 4477.16 | 4487.44 | 1958.8353 | 0.11431 | 0.10579 |
| 4487.44 | 4495 | 1958.72658 | 0.03506 | 0.02386 |
| 4495 | 4502.28 | 1958.63606 | 0.01729 | 0.01133 |
| 4502.28 | 4512.84 | 1958.52713 | 0.02161 | 0.02055 |
| 4512.84 | 4520.12 | 1958.41808 | 0.04707 | 0.03085 |
| 4520.12 | 4527.4 | 1958.32899 | 0.01777 | 0.01165 |
| 4527.4 | 4534.68 | 1958.23982 | 0.01729 | 0.01133 |
| 4534.68 | 4541.96 | 1958.15058 | 0.01537 | 0.01007 |
| 4541.96 | 4549.24 | 1958.06126 | 0.02065 | 0.01354 |
| 4549.24 | 4556.52 | 1957.97186 | 0.02017 | 0.01322 |
| 4556.52 | 4563.8 | 1957.88238 | 0.01345 | 0.00881 |
| 4563.8 | 4571.08 | 1957.79282 | 0.1023 | 0.06705 |
| 4571.08 | 4578.36 | 1957.70319 | 0.01393 | 0.00913 |
| 4578.36 | 4585.92 | 1957.61175 | 0.01201 | 0.00817 |
| 4585.92 | 4593.2 | 1957.52023 | 0.03746 | 0.02455 |
| 4593.2 | 4600.48 | 1957.43035 | 0.0365 | 0.02392 |
| 4600.48 | 4607.76 | 1957.3404 | 0.04659 | 0.03053 |
| 4607.76 | 4615.04 | 1957.25037 | 0.08982 | 0.05887 |
| 4615.04 | 4622.32 | 1957.16026 | 0.01825 | 0.01196 |
| 4622.32 | 4629.6 | 1957.07007 | 0.01489 | 0.00976 |
| 4629.6 | 4636.88 | 1956.97981 | 0.01489 | 0.00976 |
| 4636.88 | 4644.16 | 1956.88947 | 0.00913 | 0.00598 |
| 4644.16 | 4651.44 | 1956.79904 | 0.01297 | 0.0085 |
| 4651.44 | 4658.72 | 1956.70854 | 0.03026 | 0.01983 |
| 4658.72 | 4666.24 | 1956.61647 | 0.02498 | 0.01691 |
| 4666.24 | 4673.66 | 1956.52345 | 0.01345 | 0.00898 |
| 4673.66 | 4681.08 | 1956.43096 | 0.01489 | 0.00995 |
| 4681.08 | 4688.5 | 1956.3384 | 0.01297 | 0.00866 |
| 4688.5 | 4695.92 | 1956.24575 | 0.02017 | 0.01348 |
| 4695.92 | 4703.34 | 1956.15302 | 0.01729 | 0.01155 |
| 4703.34 | 4710.76 | 1956.06021 | 0.01585 | 0.01059 |
| 4710.76 | 4718.18 | 1955.96732 | 0.01105 | 0.00738 |
| 4718.18 | 4725.6 | 1955.87435 | 0.01441 | 0.00963 |
| 4725.6 | 4733.02 | 1955.78129 | 0.01441 | 0.00963 |
| 4733.02 | 4740.44 | 1955.68816 | 0.01585 | 0.01059 |
| 4740.44 | 4747.86 | 1955.59494 | 0.01537 | 0.01027 |
| 4747.86 | 4755.7 | 1955.499 | 0.02546 | 0.01797 |
| 4755.7 | 4763.12 | 1955.40298 | 0.01057 | 0.00706 |
| 4763.12 | 4770.54 | 1955.30951 | 0.01153 | 0.0077 |
| 4770.54 | 4777.96 | 1955.21596 | 0.01729 | 0.01155 |
| 4777.96 | 4785.38 | 1955.12233 | 0.01105 | 0.00738 |
| 4785.38 | 4792.8 | 1955.02863 | 0.02257 | 0.01508 |
| 4792.8 | 4800.22 | 1954.93483 | 0.01153 | 0.0077 |
| 4800.22 | 4807.64 | 1954.84096 | 0.01633 | 0.01091 |
| 4807.64 | 4815.06 | 1954.74701 | 0.01441 | 0.00963 |
| 4815.06 | 4822.48 | 1954.65297 | 0.00817 | 0.00545 |
| 4822.48 | 4829.9 | 1954.55886 | 0.02113 | 0.01412 |
| 4829.9 | 4837.32 | 1954.46466 | 0.00672 | 0.00449 |
| 4837.32 | 4845.16 | 1954.36771 | 0.0317 | 0.02237 |
| 4845.16 | 4852.58 | 1954.27068 | 0.01105 | 0.00738 |
| 4852.58 | 4860 | 1954.17623 | 0.01105 | 0.00738 |
| 4860 | 4867.42 | 1954.0817 | 0.01105 | 0.00738 |
| 4867.42 | 4874.78 | 1953.98748 | 0.02017 | 0.01337 |
| 4874.78 | 4882.14 | 1953.89355 | 0.01057 | 0.007 |
| 4882.14 | 4889.5 | 1953.79955 | 0.01249 | 0.00827 |
| 4889.5 | 4896.86 | 1953.70546 | 0.01921 | 0.01273 |
| 4896.86 | 4904.22 | 1953.6113 | 0.00384 | 0.00255 |
| 4904.22 | 4911.58 | 1953.51705 | 0.0072 | 0.00477 |
| 4911.58 | 4918.94 | 1953.42273 | 0.01009 | 0.00668 |
| 4918.94 | 4926.3 | 1953.32832 | 0.00961 | 0.00637 |
| 4926.3 | 4934.02 | 1953.28109 | 0.00865 | 0.00601 |
| 4934.02 | 4941.38 | 1953.13465 | 0.01153 | 0.00764 |
| 4941.38 | 4948.74 | 1953.04 | 0.00768 | 0.00509 |
| 4948.74 | 4956.1 | 1952.94527 | 0.03602 | 0.02387 |
| 4956.1 | 4963.46 | 1952.85046 | 0.01249 | 0.00827 |
| 4963.46 | 4970.82 | 1952.75557 | 0.0072 | 0.00477 |
| 4970.82 | 4978.18 | 1952.6606 | 0.00913 | 0.00605 |
| 4978.18 | 4985.54 | 1952.56555 | 0.01201 | 0.00796 |
| 4985.54 | 4992.9 | 1952.47042 | 0.00672 | 0.00446 |
| 4992.9 | 5000.26 | 1952.37522 | 0.03458 | 0.02291 |
| 5000.26 | 5007.62 | 1952.27993 | 0.01009 | 0.00668 |
| 5007.62 | 5014.98 | 1952.18456 | 0.04083 | 0.02705 |
| 5014.98 | 5022.7 | 1952.08677 | 0.01969 | 0.01369 |
| 5022.7 | 5030.06 | 1951.9889 | 0.01201 | 0.00796 |
| 5030.06 | 5037.42 | 1951.89329 | 0.01441 | 0.00955 |
| 5037.42 | 5044.78 | 1951.7976 | 0.00865 | 0.00573 |
| 5044.78 | 5052.14 | 1951.70182 | 0.01249 | 0.00827 |
| 5052.14 | 5059.5 | 1951.60597 | 0.01057 | 0.007 |
| 5059.5 | 5067.13 | 1951.50834 | 0.01153 | 0.00792 |
| 5067.13 | 5074.44 | 1951.41089 | 0.01249 | 0.00822 |
| 5074.44 | 5081.75 | 1951.31545 | 0.00913 | 0.00601 |
| 5081.75 | 5089.06 | 1951.21992 | 0.0317 | 0.02086 |
| 5089.06 | 5096.37 | 1951.12432 | 0.00672 | 0.00443 |
| 5096.37 | 5103.68 | 1951.02864 | 0.00865 | 0.00569 |
